# Cortical topographic motifs emerge in a self-organized map of object space

**DOI:** 10.1126/sciadv.ade8187

**Published:** 2023-06-21

**Authors:** Fenil R. Doshi, Talia Konkle

**Affiliations:** Department of Psychology and Center for Brain Sciences, Harvard University, Cambridge, MA, USA.

## Abstract

The human ventral visual stream has a highly systematic organization of object information, but the causal pressures driving these topographic motifs are highly debated. Here, we use self-organizing principles to learn a topographic representation of the data manifold of a deep neural network representational space. We find that a smooth mapping of this representational space showed many brain-like motifs, with a large-scale organization by animacy and real-world object size, supported by mid-level feature tuning, with naturally emerging face- and scene-selective regions. While some theories of the object-selective cortex posit that these differently tuned regions of the brain reflect a collection of distinctly specified functional modules, the present work provides computational support for an alternate hypothesis that the tuning and topography of the object-selective cortex reflect a smooth mapping of a unified representational space.

## Teaser

Self-organizing principles provide a computational account for the topographic organization of the high-level visual system.

## INTRODUCTION

Extensive empirical research has charted the spatial layout of tuning preferences along the ventral visual stream [occipitotemporal cortex “OTC” in humans and inferior temporal “IT” cortex in monkeys; for review, see (*1*–*3*)]. At a macro-scale, there are two major object dimensions that have been shown to elicit systematic large-scale response topographies, related to the distinction between animate and inanimate objects (*4*–*7*) and the distinction between objects of different real-world sizes (*8*–*10*). Further research has shown that these seemingly high-level animacy and object size distinctions are primarily accounted for by differences in tuning along more primitive visuo-statistical features that meaningfully covary with these high-level properties [e.g., at the level of localized texture and coarse form information; (*11*–*13*)].

At a mesoscale, there is a hallmark mosaic of category-selective regions scattered across this cortex, defined by their spatially clustered and highly selective responses to a particular category—e.g., faces, bodies, letters, and scenes (*2*, *14*–*23*)—with no such highly selective regions for other categories like cars and shoes (*24*). Initially, it was unclear whether these regions should be considered “stand-alone modules,” which are unrelated to the object-tuning preferences of the surrounding regions (*25*). However, it is increasingly clear that there is a systematic encompassing structure in the cortical organization, where the face-, body-, and scene-selective regions fall systematically and meaningfully within this larger-scale animacy and object size organization (*1*, *9*, *26*). This systematic map of object tuning, at both macro- and mesoscales, has led to an extensive debate and discussion—why are these macro- and mesoscale object distinctions evident and not others, and why are they spatially organized this way (*1*–*3*, *8*, *27*–*29*)?

On one theoretical account, the tuning and topography of neurons in the object-selective cortex could be conceived of as jointly capturing an integrated representational space, which is smoothly mapped along the cortical surface (*9*, *26*). That is, the tuning of each neuron in this population is best understood together, as part of a large-scale population code, with features designed to discriminate all kinds of visual input, including faces (*30*–*32*). This account maintains that this multidimensional representational space is mapped along the two-dimensional (2D) cortex such that similar tuning is nearby, and more distinct tuning is farther apart (*1*, *33*, *34*). On this account, animacy and object size distinctions have a large-scale organization because they are related to the major dimensions of this unified visual feature space. At the same time, mesoscale regions for faces, bodies, and scenes emerge due to their related visuo-statistical characteristics with other object categories, without requiring other specialized mechanisms.

This integrated account of the tuning and topography of the object-selective cortex has been challenging to test, as there were no image-computable feature spaces rich enough to categorize many kinds of objects (*35*). However, deep neural networks (DNNs) trained to do many-way object categorization, without any special feature branches set aside for some categories, provide precisely this kind of representational space (*36*, *37*). Recently, Bao *et al*. (*26*) used a late layer of a DNN (AlexNet) to operationalize such a unified representational space, proposing that the monkey IT organization can be thought of as a coarse map of this space. In so doing, they could predict the tuning of previously uncharted regions of the primate visual cortex based on the major dimensions of the DNN feature space, and they linked animacy and object protrusion distinctions to the major principal components of this DNN space. Relatedly, Huang *et al*. (*38*) have found that information about the real-world size of objects is encoded along the second principal component of the late stages of DNNs. Furthermore, Vinken *et al*. (*39*) recently demonstrated that face-selective neurons in IT could be accounted for by the feature tuning learned in these same object-trained DNNs; also see (*36*, *37*, *40*). Thus, DNNs clearly operationalize a multidimensional representational encoding space that has information about these well-studied object distinctions.

One critical missing component of this theoretical account, though, is how to bridge from the multidimensional representational spaces of DNNs to the spatialized tuning of the cortical sheet—that is, to have a computational account of not only what the tuning is but also where it is located on a 2D surface. Concurrently, a variety of approaches are emerging to bring spatial organization in DNNs, all of which operate at different levels of abstraction regarding the underlying mechanisms (*41*–*44*). Here, we cast the problem of topography as one of data-manifold mapping, leveraging Kohonen self-organizing maps (SOM) (*45*). This computational approach aims to reveal the similarity structure of natural images implicit in the DNN feature space, by smoothly embedding a 2D sheet into the multidimensional feature space to capture this structure. This computational approach has previously been successfully used to account for other representational-topographic signatures found along the cortex, including the large-scale multiple-mirrored map topography of the early visual system areas (*46*–*48*), the large-scale body-part and action topography of the somatomotor cortex (*49*–*51*), and even early explorations of object category topography (*34*).

We developed a framework to train a SOM over the feature space learned in the late stage of a DNN model, and then probed for several key signatures of the ventral stream topography. Doing so revealed several brain-like macro- and mesoscale response topographies, which naturally emerge from a smooth mapping of the DNN feature space, including the formation of localized category-selective regions for faces and scenes. However, not all known topographic signatures of the ventral visual pathway were evident in the modeled topography. Broadly, this work provides computational plausibility for a theoretical account in which the organization of object-selective cortex can be understood as a smooth mapping of a unified representational space along a 2D sheet. Further, under these assumptions, the departures between the object representation in DNNs and the human brain reveal clear modeling directions to drive toward a more brain-like representational system.

## RESULTS

### Learning the data manifold of a deep neural network feature space using self-organizing maps

**Fig. 1.**
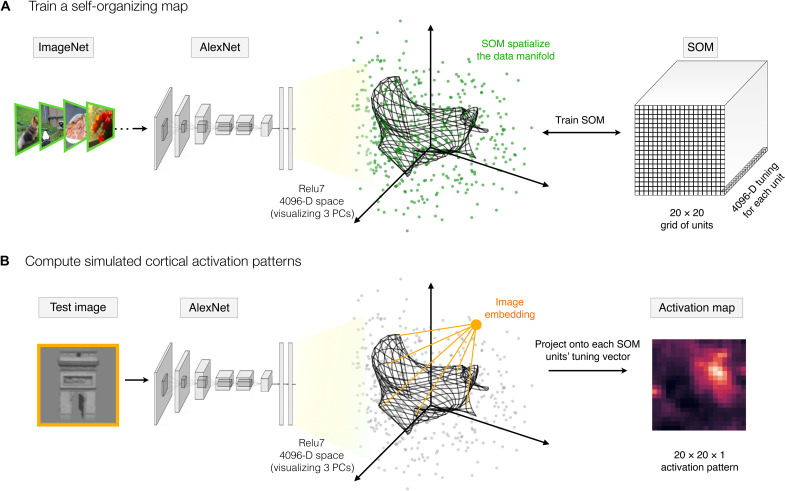
Self-organizing the features space of a deep neural network. (**A**) A self-organizing map is appended to a pretrained AlexNet, following the relu7 stage. The relu7 layer is a 4096-D feature space, visualized here along the first three principal components (PCs), where the green dots reflect the embedding of a sample of ImageNet validation images. The final SOM layer consists of a 2D map of units of size 20 × 20, each with 4096-D tuning (depicted as a black grid). During training, the tuning curves of these map units are updated to capture the data manifold of the input images (i.e., the set of green dots). (**B**) To compute the spatial activation map for any test image, the image is run through the model and the relu7 embedding is computed. Then, for each map unit, the projection of the image embedding onto the tuning vector is computed (conceiving of these tuning vectors as carrying out a filter operation), and this value is taken as the activation of this map unit to this image.

Here, we use a standard pretrained AlexNet neural network (*52*), focusing on the representation of natural images in the penultimate layer (relu7) before the output layer. This stage reflects the most transformed representational format from the pixel-level representation. Within this layer, the set of natural images is represented in a 4096-D space, which we visualize in Fig. 1A along the first three principal components for a sample of 500 images. Within this multidimensional space, some images are nearby—eliciting similar activation profiles across the set of DNN units, while other images are farther apart— eliciting more distinct activation profiles. The set of all natural images in this space comprises the data manifold.

Next, we add a SOM layer, which can be conceived of as an additional fully connected layer, where the tuning of each unit of the SOM is a weighted combination of the relu7 features. These tuning vectors of SOM units are trained with the goal of smoothly capturing the data manifold. Specifically, the algorithm projects a 2D grid of units into the relu7 space, learning tuning curves for each unit such that units with nearby tuning in the relu7 representational space are also spatially nearby in the grid of map units. Furthermore, the algorithm is designed to ensure that the collective set of map units has close coverage over the entire data manifold. Thus, if there are parts of this feature space that are occupied by natural images, there will be some map units tuned near that part of the representational space. And, if there are combinations of relu7 feature activations that no natural images ever activate, then no SOM units will have tuning curves that point to that part of the representational space. In this way, the SOM transforms the implicit representation of natural images embedded in the feature space to be an explicit map of the data manifold.

The SOM was trained with an iterative algorithm, following standard algorithm (*45*) procedures (see Materials and Methods for details). Note that the specifics of the learning algorithm are not intended to be interpreted as a direct mechanistic model of cortical development. To overview, first, the tuning of each SOM unit was initialized in a grid covering the plane of the first two principal dimensions of the relu7 feature space. Next, the tuning of each unit was iteratively and competitively updated to be increasingly closer to the input data samples while also ensuring that neighboring units in the map are updated toward similar parts of the data manifold. Here, the 50,000 images from the validation set of ImageNet (*53*) were run through a pretrained AlexNet (with no additional DNN weight updates), and the activations from the relu7 stage were used as the input data distribution to train the SOM layer. Additional details related to SOM initialization, neighborhood parameters, learning rate, and other parameters guiding the training process are detailed in Materials and Methods. At the end of the training, the resulting layer is referred to as a SOM or a map, which consists of a grid of units (here 20 × 20), each with a 4096-D tuning curve.

Figure 1A provides a graphical intuition, where the tuning of each map unit is projected into the feature space, with SOM map units depicted as a grid of connected points. Here, the tuning of the units on the SOM (i.e., their locations in this feature space) is shown at an intermediate stage of the training, for clarity. Figure S1 visualizes the SOM at different training stages from initialization to final. Figure S2 plots the quality of the fit of the SOM to the input data as a function of training epochs, as well as the final tuning similarity between all pairs of SOM units as a function of distance on the trained map.

We next established a pipeline to measure a spatial activation profile over the output map, for any given test image (Fig. 1B). To do so, we pass an image through the pretrained AlexNet to compute its 4096-D vector in the relu7 space. Then, we compute the response of each SOM unit by conceiving it as a filter, where the activation of each unit is computed based on the tuning-weighted combination of feature activations (see Materials and Methods). With these procedures in place, we next followed the empirical literature, leveraging the same stimulus sets and analysis techniques used to map the response topography of the ventral visual stream, but here computed over the simulated activations of the SOM. Any emergent tuning and topography of object distinctions are thus present in the implicit similarity structure of the DNN representation.

### Large-scale organization of animacy and real-world size

**Fig. 2.**
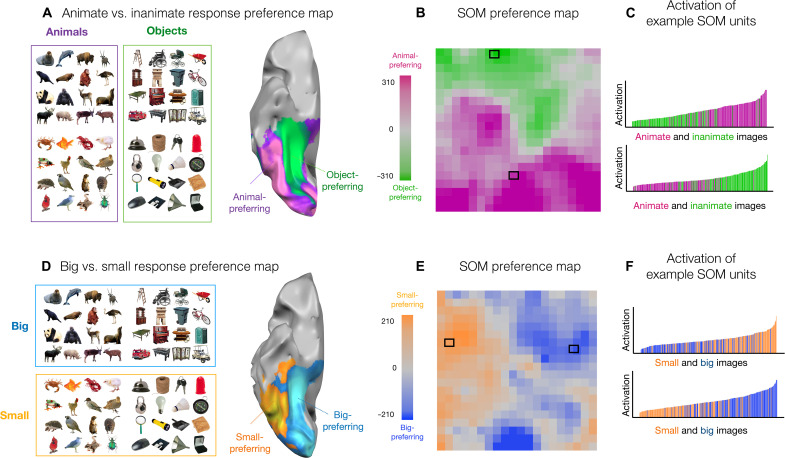
Large-scale organization of animacy and size. (**A**) Example images of animals and objects are shown adjacent to the corresponding brain preference map. A ventral view of a partially inflated hemisphere is shown where regions with stronger responses to depicted objects are shown in green and stronger responses to depicted animals are shown in purple. (**B**) Each unit of the simulated cortex is colored by its response preference to either animal or object images. (**C**) Two units were selected that show the maximum distinction (computed using *t* tests) between animate and inanimate objects. For both of these units, the degree of activation (*y* axis) is plotted for all 240 localizer images (*x* axis), sorted by their activation. (**D**) Same images as in (A) but now grouped by whether they depict big or small entities in the world adjacent to the corresponding brain preference map. (**E**) Each unit of the simulated cortex is colored by its response preference for images of big or small entities. Stimuli and brain maps adapted from (*9*). (**F**) Two units were selected that show the maximum distinction (computed using *t* tests) between small and big entities. For both of these units, the degree of activation (*y* axis) is plotted for all 240 localizer images (*x* axis), sorted by their activation.

We first tested for the representational distinction between animate versus inanimate objects. Stimuli from (*9*) were used, which depict animals and inanimate objects in color on isolated backgrounds (120 each; see examples in Fig. 2A). Response preferences along the ventral surface of the brain show a large-scale organization by animacy—that is, with an extensive swath of cortex with higher activations to depictions of animals (purple), adjacent to an extensive swath of cortex with higher activations to inanimate objects (green); data from (*9*).

For each SOM map unit, we measured the average activation to these same images of animals and objects and visualized the degree of response preference along the simulated cortical sheet (see Materials and Methods). The results are shown in Fig. 2B. Each map unit is colored by whether it has a stronger response to depicted animals or inanimate objects, with stronger response preferences depicted with deeper color saturation. We find that the distinction between animals and inanimate objects reveals many units with preferences for either domain, clustered at a relatively large scale across the entirety of the map. Such an organization was not present when applying the SOM on the same layer’s feature space in an untrained DNN, nor in a SOM that was randomly tuned in a 4096-D feature space (fig. S3). This organization was also not present in a SOM directly trained in a pixel space representation (fig. S4; see the Supplementary Materials).

A second factor that yields large-scale topographic distinctions along the cortical surface of the human ventral visual stream is that of real-world size, shown in Fig. 2D (*8*–*10*). That is, there is an extensive swath of cortex that responds more to depicted entities that are typically big in the world (e.g., chairs, tables, landmarks, body-sized, or bigger) and an adjacent swath of cortex that responds more to depicted entities that are typically small in the world (e.g., shoes, mugs, tools, and other hand-held manipulable objects), even when these images are presented to the observer at the same visual size.

To visualize the topography of real-world size preferences across the SOM, the same stimuli from (*9*) were used, but instead grouped by size. The size preference map of the SOM again shows a relatively large-scale organization of this factor, with map units showing stronger activations to either big or small entities, clustered at a relatively large scale across the entirety of the map (Fig. 2E). This large-scale organization of response preferences was not present when applying the SOM on the same layer’s feature space in an untrained DNN, nor in a SOM that was randomly tuned in a 4096-D feature space, nor in a model directly trained in the pixel space representation (figs. S3 and S4; see the Supplementary Materials).

These analyses reveal that the distinctions between depicted animate and inanimate objects, and between big and small entities, are related to the major factors of the feature space learned in the DNN. For example, it could have been the case that units with animal and object response preferences were tightly interdigitated or that there were many map units with relatively weak response preferences and only a few with strong domain preferences. Previous empirical work has clearly demonstrated that the animate/inanimate distinction is known to be a major factor in the geometry of both human and nonhuman primate representation along the ventral stream (*54*); here, the SOM reveals this property of the DNN representational structure in a spatialized format, as a large-scale organization of the response landscape.

### The role of mid-level visual feature differences in animacy and real-world size organizations

Although different regions of the brain are systematically activated by images of animals or objects of either big or small sizes, this result does not therefore directly imply that these map units are driven by something very abstract about what it means to be animate or inanimate, big or small. Rather, increasing empirical evidence indicates that responses along this purportedly “high-level” visual cortex have a substantial degree of tuning at a more primitive visuo-statistical level (*11*–*13*, *55*). To this end, the next signature of ventral stream topography that we probed is its sensitivity to images with more primitive “mid-level” image statistics preserved (*11*).

**Fig. 3.**
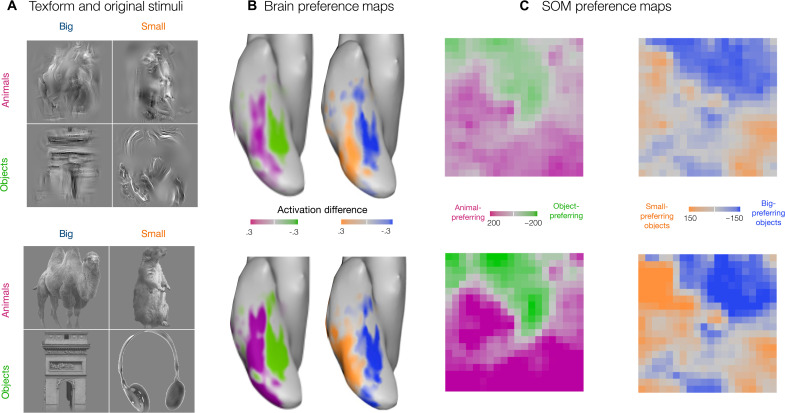
Sensitivity to mid-level featural distinctions. (**A**) Texform images (top) generated using a texture synthesis algorithm from recognizable images (bottom) of 30 big objects, 30 small objects, 30 big animals, and 30 small animals. (**B**) Preference maps for animacy and size for stimuli shown in (A) along the OTC. The limits of the color bar reach full saturation at an absolute value of 0.3 reflecting the beta difference computed from an individual’s GLM. (**C**) Preference maps for animacy and size on the simulated cortex, for texform and original stimuli. Each unit of the simulated cortex is colored based on their preference for animacy, i.e., animals versus objects and for size, i.e., big versus small objects (purple for animals and green for objects in the animacy map and orange for small objects and blue for big objects). Stimuli and brain maps are adapted from (*11*).

Long *et al*. (*11*) created images using a texture synthesis algorithm (*56*), which preserved local texture and coarse form information of the original animal and object images, but which were sufficiently distorted to be empirically unrecognizable at the basic level (e.g., lacking clear contours, 3D shape; example stimuli are shown in Fig. 3A) (*11*). However, these “texform” images still evoked systematic and topographically organized responses along even the later stages of the ventral visual stream. Furthermore, the cortex with a preference to animate versus inanimate recognizable stimuli showed the same large-scale organization in response to texforms, as shown in Fig. 3B. The same holds for real-world size.

To test for these signatures in the SOM, we used the same stimulus set as in the neuroimaging experiment, which consisted of 240 grayscaled, luminance-matched images (120 originals and 120 texforms, each with 30 exemplars from big, small, animate, and inanimate objects). Figure 3C shows the corresponding preference maps for texform images and original images, for both animals versus objects and big object versus small object contrasts. We find that the mid-level image statistics preserved in texforms are sufficient to drive near-identical large-scale organizations across the SOM (correlation between original and texform maps: animacy *r* = 0.93, *P* < 10^−5^; size *r* = 0.85, *P* < 10^−5^).

Thus, these results provide further corroborative evidence that it is possible to have a large-scale organization that distinguishes animals from objects and big objects from small objects without requiring highly abstract (non-visual) features to represent these properties. Instead, this seemingly high-level organization can emerge from visuo-statistical differences learned by DNNs that are particularly reliant on coarsely localized textural features.

### Category selectivity for faces and scenes

**Fig. 4.**
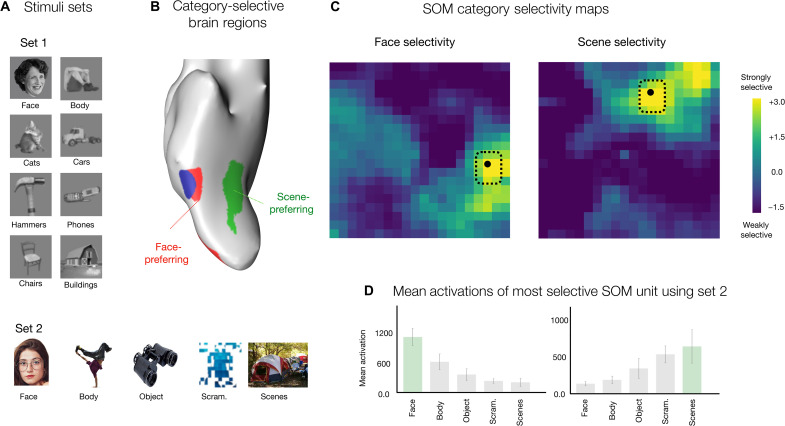
Face and scene selectivity. (**A**) Example images from two stimuli sets: stimuli set 1 containing luminance-matched grayscale images from eight different categories—faces, bodies, cats, cars, hammers, phones, chairs, and buildings; stimuli set 2 containing colored images from five different categories—faces, bodies, objects, scenes, and scrambled images—on a white background. (**B**) Ventral view of the inflated cortical surface of one individual, highlighting a face-selective region in red, a body-selective region in blue, and a scene-selective region in green. (**C**) Face selectivity and scene selectivity maps are shown, reflecting a d-prime measure computed over responses to images from stimuli set 1. Each unit is colored based on its selectivity for the target category versus the remaining categories. (**D**) The most face- and scene-selective map unit was identified, and responses were measured for independent images from stimulus set 2. Bar plots show the mean activations of the face-selective map unit (left) and scene-selective map unit (right). Stimulus sets were from (*59*) and (*9*); and brain maps are adapted from (*9*).

Seminal early findings of ventral visual stream organization also discovered and mapped a small set of localized regions of the cortex that have particularly strong responses for some categories of stimuli relative to others, e.g., for faces, scenes/landmarks, bodies, and letter strings (e.g., see Fig. 4B) (*15*–*20*, *22*, *23*, *57*). Some theoretical accounts of these regions consider these as independent and unrelated functional modules, implicitly assuming no direct relationship between them (*2*, *58*). However, the integrated feature space of the DNN allows us to consider an alternate hypothesis that face and scene selectivity might naturally emerge as different parts of a common encoding space—one whose features are designed to discriminate among all kinds of objects more generally (*9*, *26*, *36*, *37*, *39*). If this is the case, then these categories would drive responses in a localized part of the feature space, which would emerge as a localized cluster of selective responses in the SOM.

To explore this possibility, for each map unit, we measured its mean response to images from two different localizer sets [stimulus set 1: grayscaled luminance-matched images of faces, bodies, cats, cars, hammers, phones, chairs, and buildings; 30 images per category; see example images in Fig. 4A (*59*); stimulus set 2: 400 color images of isolated faces, bodies, objects, scenes, and scrambled objects on a white background, 80 images per category (*9*); see example images in Fig. 4A]. Next, for each unit, we calculated the selectivity magnitude, a measure of the d-prime score reflecting the difference between, for example, the response magnitude for all face images, compared with the response magnitude for all nonface images from the set (see Materials and Methods).

Figure 4C plots the selectivity maps for both face and scene selectivity measures, computed over stimulus set 1. We find that there are map units with relatively strong selectivity to faces and scenes, clustered in different parts of the SOM. These units showed strong categorical separability (e.g., all face images within the image set were the strongest activating images for the most face-selective unit, while all building images were the strongest activating images for the most scene-selective unit). As a further test of generalizability, we measured the response of the most face- and scene-selective units in the map to an independent stimulus set, which has different image characteristics. These units again show the strongest response to their preferred category (Fig. 4D). The same results were obtained with an alternative selectivity index (SI) metric for computing category selectivity (fig. S5).

These analyses demonstrate that face and scene regions can naturally emerge in a smoothly mapped DNN feature space, one whose features are learned in service of discriminating many kinds of objects. Thus, these results provide computational evidence for a plausible alternative to the theoretical position that distinct, domain-specialized mechanisms are required for specialized regions with category selectivity to emerge.

### Macro- and mesoscale organization

In the human brain, there is a systematic relationship between the locations of the mesoscale category-selective regions and the response preferences of the surrounding cortex (*1*, *9*). Specifically, the face-selective regions fall within and around the larger zones of the cortex that have a relatively higher preferential response to depicted animals, while scene-selective regions fall within zones of the cortex that have a relatively higher preferential response to depicted inanimate objects. In the simulated cortex, we find that the same topographic relationship naturally emerges.

**Fig. 5.**
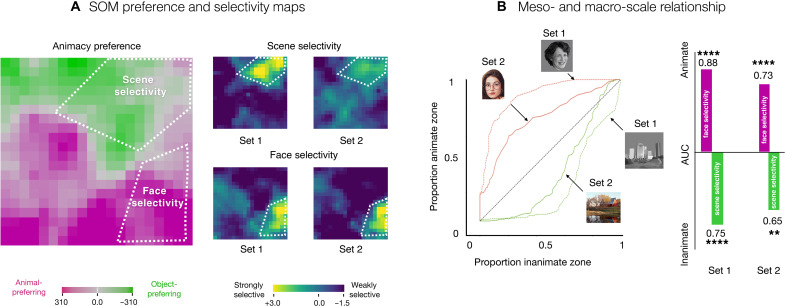
Relationship between macro- and mesoscale organization. (**A**) Preference and selectivity maps. White lines visually demonstrate where the most face-and scene-selective regions fall in reference to the animacy preference zones on the simulated cortex. (**B**) Area under the curve (AUC) analysis to quantify the category-selective overlap with the preference zones. On the left, we see receiver operating characteristic (ROC) curves for faces and scenes for both stimuli sets. These curves reflect how each of the preference zones fills up as an increasing number of map units on the SOM get included, starting from the most selective. On the right, we compute AUC for the ROC curves. Significance is based on permutation tests: **P < 10^−2^; ****P < 10^−4^.

Figure 5A shows the SOM animate versus inanimate preference map, alongside maps of face and scene selectivity, computed for the two different stimuli sets. Qualitative inspection reveals that units with the strongest face selectivity are located within the region of the map with animate-preferring units and units with the strongest scene selectivity are located within the region of the map with inanimate-preferring units.

To quantify the relationship between category-selective maps and the animate-inanimate preference maps, there is a challenge of what threshold to pick to define a “category-selective” region to compute its degree of overlap with the animate-preferring and inanimate-preferring units. To circumvent this issue, we used a receiver operating characteristic (ROC) analysis, following the procedures used in (*9*); see Materials and Methods. This method sweeps through all thresholds and quantifies where the most selective face units are located, as a proportion of whether they fall in the animate or inanimate zones. By varying the selectivity cutoff threshold (from strict to lenient), this method traces out an ROC curve between (0,0) and (1,1), where the area between this curve and the diagonal reflects how strongly the most selective map units falls within one zone (or the other). Specifically, Fig. 5B plots the ROC curves and area under the curve (AUC) measures. The face-selective units mainly fall in the animate zones (set 1: animate AUC = 0.88, *P* < 10^−5^; set 2: animate AUC = 0.73, *P* < 10^−5^), while the scene-selective units within the inanimate preferring zone (set 1: inanimate AUC = 0.75, *P* < 10^−4^; set 2: inanimate AUC = 0.65, *P* < 10^−2^).

These analyses over the SOM recapitulate previous findings in the brain, highlighting the systematic situation of category-selective units within the context of the large-scale organization. Hence, they provide computational plausibility for the theoretical position that, in the human brain, category-selective regions are not independent islands but, instead, are meaningfully related to each other and to the less-selective cortex just outside them, as part of a unified representational space.

### Divergence between brain and model response topographies

While we have emphasized the topographic signatures that converge between the organization of the human object-responsive cortex and the SOM of the penultimate AlexNet layer, there are also clear cases of divergence, at both macro- and mesoscales. Specifically, these differences are evident when considering (i) the interaction between animacy and real-world size properties and (ii) considering which categories show more localized versus distributed selectivity.

The first major difference is related to the way the feature tuning of the DNNs spans the animacy and object size distinctions, compared to the human brain. In the simulated cortex, the animacy and object size organizations are relatively orthogonal, e.g., Fig. 2B shows animate-to-inanimate preferences from the bottom to top of the SOM, and Fig. 2E shows small-to-big preference from left to right of the SOM. In contrast, as can be seen in the brain organizations in Fig. 2 (A and D), both the inanimate-to-animate and big-to-small contrasts actually evoke a very similar spatial organization along the ventral visual stream, with preferences that both vary from medial to lateral.

**Fig. 6.**
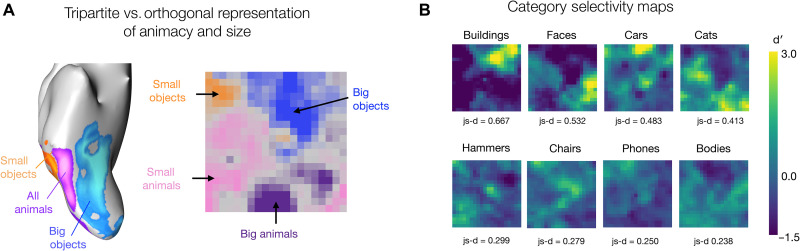
Divergences between the brain and the SOM. (**A**) Left: Three-way preference map in the OTC among big objects, all animals, and small objects; adapted from (*9*). Right: Four-way preference map in the SOM among big objects, small objects, big animals, and small animals using the same stimuli as used in (*9*). (**B**) Category selectivity maps computed using the d′ measures with each of the eight categories from the stimulus set used in (*59*) serving as the target, and the other seven categories serving as the nontarget images. These plots are organized by an approximate estimate of their nonuniformity, calculated with a js-distance score reflecting on how strongly the distribution of selectivity scores deviates from a distribution of uniform selectivity.

Konkle and Caramazza (*9*) delineated how these two organizations fit together in the human brain, revealing a “tripartite” organization of object tuning (Fig. 6A). Specifically, they observed that there are three parallel zones of cortex with stronger responses for either depicted big objects, all animals (independent of size), and small objects. Put another way, big and small animals activated relatively similar large-scale patterns across the cortex. The SOM, in contrast, shows an organization with clearer four-way separability among these conditions (Fig. 6A). That is, there are zones of SOM map units with a relatively stronger response to either small objects, big objects, small animals, or big animals. This lack of tripartite structure is also evident in the representational geometry of the deep net (fig. S6A), highlighting that this divergence is not an artifact of the self-organization process but is inherently present in the structure of the deep net feature space itself.

The second divergence between the cortical topography and the SOM of the DNN feature space is related to category-selective signatures across different categories. In the human brain, no highly selective and circumscribed regions have been mapped for cars, shoes, or other categories (*24*). However, in the simulated cortex, there is a different pattern. Figure 6B shows selectivity maps for each of the eight categories in the first stimulus set, computed as the d-prime score between the responses over the target category images, relative to the responses over the nontarget category images. Qualitative inspection shows that the SOM does not have strongly localized selectivity for bodies, while it does show localized selectivity for cars (and, to some extent, cats).

In a subsequent post hoc analysis, we found that body selectivity was more evident when excluding faces from the d-prime calculation; doing so reveals units with higher body selectivity located precisely where the face-selective units are (fig. S7). Furthermore, images of faces and bodies are the maximally activating images for neighboring units on the SOM grid (true across several stimulus sets; see fig. 8, C and D), consistent with the anatomical proximity of face- and body-selective regions of the human brain (*60*, *61*). Thus, body and face tunings are in similar parts of the feature space but are less separable in the SOM than is evident in cortical organization. Together, these examples reveal that the DNN feature space, when smoothly mapped, has some of representational-topographic signatures that do not perfectly align with the response structure of the object-selective cortex in the human brain.

### A map of object space

The analyses of the tuning of units on the SOM thus far have focused on activation landscapes to different stimulus conditions, similar to the approach taken in functional magnetic resonance imaging (fMRI) and other recording methods, which measure and compare brain responses to targeted images. However, the tuning of each map unit in the SOM is specified in a feature space of a DNN that is end-to-end differentiable with respect to image inputs. This enables us to leverage computational synthesis techniques to visualize the tuning across the map (*62*). Specifically, for each unit’s tuning vector, we extract derivatives with respect to the image and iteratively adjust the pixel values (starting from a noise seed image) such that it maximally drives a specific unit of the SOM (see Materials and Methods).

**Fig. 7.**
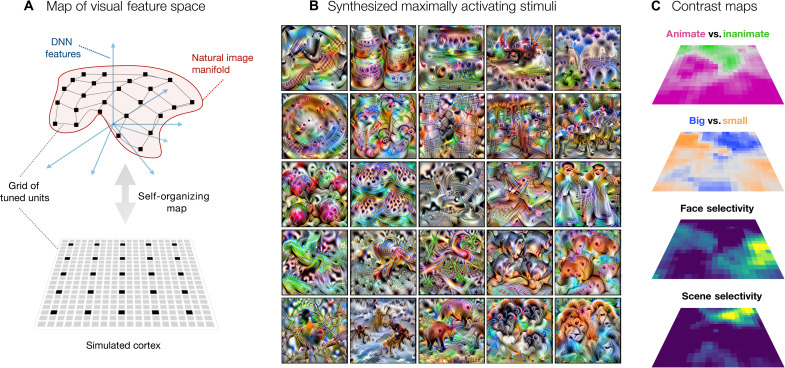
A map of object space. (**A**) Manifold of natural images formed by the images’ DNN features shown in red. The axis demonstrates each of the 4096 dimensions in the relu7 activations of the DNN. The gray connected lines depict the simulated cortex trying to hug this image manifold and the black points depict units on the map/simulated cortex. This simulated cortex can be described via a 2D grid of connected units, i.e., the SOM. (**B**) Images synthesized using gradient ascent to maximally activate the map units highlighted in (A) via black points. (**C**) Entire spatial hierarchy learned by the simulated cortex—animacy and size preference and face and scene selectivity.

Figure 7A schematizes the SOM, embedded in the high-dimensional feature space of the DNN representational space, and depicted below as a flattened grid of tuned units. For a subset of units systematically sampled across the map (25 units highlighted in black), Fig. 7B shows the corresponding synthesized image that maximally drives these units. Figure S9 shows the synthesized images for all the map units on the SOM. At a glance, these images seem to capture rich textural features, consistent with what is now known about the nature of the feature representations in DNNs (*63*, *64*). A more detailed inspection shows that the nature of the image statistics captured across the map varies systematically and smoothly, e.g., with synthesized maximally activating images that clearly are more animal-like or more scene-like in different parts of the map. As a complementary visualization, in fig. S8, we show the image that maximally drives each map unit, computed over different stimulus sets, including those from (*26*).

Figure 7C provides further context for understanding the map of object space, showing how the organizations of animate versus inanimate, big versus small entities, face selectivity, and scene selectivity, all are evoked from the same spatialized feature space. This visualization further helps clarify how these preferences for animate versus inanimate objects, big versus small entities, and localized regions for faces and scenes can be related purely to different image statistics (as any more abstract, nonvisual level of representation is beyond the scope of this DNN).

### Additional analysis

We conducted several SOM variations to examine the robustness of these representational-topographic motifs. Figure S10 shows little to no effect of changing or increasing the number of images used to initialize the SOM tuning. Figure S11 shows that SOMs with approximately two to three times more units also showed the same motifs.

Finally, here, we focused on an AlexNet model architecture, trained on the Imagenet dataset, trained with 1000-way categorical supervision. However, this work also introduces a general method of using SOMs to visualize the impact of different input datasets, architectures, and objectives in shaping the format of the learned representation (*65*).

As one initial step to this end, we explored the organization of the same AlexNet model, trained instead on the Ecoset database (*66*). This model experiences a different distribution of visual images and categories—including fewer animal categories. We mapped the penultimate layer representation using the same SOM procedures. The resulting large-scale SOM topography was quite similar but did show slightly more tripartite structure for the animacy and object size dimensions (fig. S6B). This analysis highlights that the visual experience (i.e., image set curation) does subtly alter the learned representational space and resulting topographic organization, though this particular diet alone was not sufficient to lead to markedly more brain-like topographic motifs (e.g., still no body selective regions).

As a second analysis, we explored the organization of a similar AlexNet model architecture experiencing the ImageNet dataset but trained instead with self-supervised objectives [e.g., Instance-Prototype Contrastive Learning (IPCL) and Barlow Twins; (*67*, *68*)]. These image-level objectives are designed to learn features that support fine-grained distinctions among all visual input, without presupposing or requiring any category information, and are known to yield learned representational spaces with notable similarity to category-supervised objectives (*69*), with comparable levels of brain predictivity (*67*, *70*). We also found similar topographic motifs naturally emerged in these self-supervised models, following the same SOM procedures (figs. S12 and S13). However, qualitatively, the organizations and patterns in the data are less clear-cut than the category-supervised model. We save the task of a deeper detailed analytical comparison for future work. For the present work, these self-supervised objectives provide initial evidence for an even stronger argument that these brain-like representational distinctions and topographic motifs can arise without requiring any external categorical pressures to shape the visual feature space.

## DISCUSSION

Here, we used a SOM algorithm to spatialize the representational structure learned within the feature space of a DNN trained to do object categorization. This method yields a 2D grid of units with image-computable tuning that reflects a smooth mapping of the data manifold in the representational space. We tested whether several hallmark topographic motifs of the human object-responsive cortex were evident in the map, finding several convergences. First, large-scale divisions by animacy and real-world object size naturally emerged. Second, the same topographic organizations were elicited from unrecognizable “texform” images, indicating that the feature tuning is sensitive to mid-level visual statistical distinctions in these images. Finally, clustered selectivity for faces and scenes naturally emerged, without any specialized pressures to do so, and was situated systematically within the broader animacy organization, as in the human brain. However, the simulated cortex did not capture all macro- and mesoscale signatures. For example, it contained an orthogonal rather than a tripartite representation of animacy and size and lacked localized body-selective regions, leaving open questions for what is needed to learn an even more brain-like organization. Theoretically, this work provides computational plausibility toward a unified account of visual object representation along the ventral visual stream.

### Implications for the biological visual system

After two decades of functional neuroimaging research charting the spatial structure of object responses along the ventral visual stream, it is clear that there is a stable, large-scale topographic structure evident across people; however, the guiding pressures that lead to this stable organization are highly debated (*25*, *28*, *71*–*75*). On one extreme, for example, the nature of the tuning and the locations of category-selective regions are primarily driven by specialized pressures that are innate and nonvisual in nature, with supporting evidence from distinct long-range connections beyond the visual system and colocalized functional activations in the congenitally blind (*73*, *74*, *76*–*78*). On the other extreme, it is the experienced statistics of the visual input, scaffolded from an initial retinotopic organization and generic learning mechanisms, that are primary drivers of the organization in the object-selective cortex (*8*, *75*, *79*–*81*). What can the present modeling work contribute to this debate?

Here, we suggest that, by probing the representational signatures evident in this model, we gain traction into what kind of object distinctions can emerge from the experienced input, without requiring category-specialized pressures. That is, the network is capable of extracting the regularities in input distributions, reformatting them into a code that can support downstream behavior-like object categorization. For example, the AlexNet architecture we used does not have any explicit learning mechanisms devoted to some special categories [e.g., branching architectures that are trained only with faces; (*82*)]. Similarly, the SOM also does not have any category-specific learning rules. In this way, our model leverages a relatively generic set of inductive biases that guide the structure of the learned visual feature space. In this way, rather than thinking of this DNN as an exact model of the visual system, we can think of it instead as a functionally powerful representation learner.

On this framing, the fact that the SOM shows a large-scale organization by animacy and object size, without explicit connectivity-driven pressures or domain-specific learning mechanisms that enforce these groupings, means that these “high-level” distinctions can emerge directly from visuo-statistical differences in the input. The results with texforms corroborate this interpretation. Critically, these organizations were not present in the pixel space or in the late stages of untrained DNNs, which highlights that the visuo-statistical properties underlying animacy and object size distinctions are a consequence of the hierarchical untangling of the DNN. Furthermore, we show that even clustered face selectivity and scene selectivity emerge—indicating that depicted faces and scenes have particularly a focal and separable location in the DNN feature space—and need not be attributed to specialized learning pressures. Certainly, this result does not provide direct mechanistic evidence for the experience-based formation of these regions in the brain. But while experience-based accounts formerly could only speculate that certain object category distinctions could emerge from input statistics alone, this work now provides clear support for the sufficiency of image statistics to form a basis for the emergence of these distinctions.

Finally, it is important to acknowledge that there are also many other empirical signatures of object topography, which these models are not yet directly equipped to test. For example, object topography along the cortex in humans is “mirrored,” with duplicated selectivity on the ventral and lateral surfaces (*75*, *83*, *84*). This duplication has been hypothesized to emerge from extensions of adjacent retinotopy, reflecting the divisions of the upper and lower visual field [though the influence of nonduplicated motion area (MT) on the lateral surface has also been hypothesized]. More generally, there is an extensive trove of empirical and anatomical data, coupled with existing hypotheses about their role in driving the tuning and topography along the ventral visual stream, simply awaiting the advancing frontier of image-computable modeling frameworks to explore these theories. Until then, we offer that considering this DNN model and SOM as a representational system, rather than a direct model of the visual system, still allows for computational insights into the possible pressures guiding the organization of the ventral visual stream.

### Modeling cortical topography

How does the approach taken here relate to concurrently developed techniques bringing spatialized responses to DNNs (*41*–*44*)? Across the set of approaches, all seem to be conceiving of the problem at different levels of abstraction, and test for different signatures. For example, Lee *et al*. (*41*) conceive of the early convolutional layers as already having topographic constraints, while the fully connected layers do not; they arranged the fully connected units in a grid and added a spatial correlation loss over the tuning during the model training, in addition to the object categorization objective. They found clusters of face-selective units that were connected across the fully connected layers—they did not, however, probe for animacy, object size, or other category-selective regions. Blauch *et al*. (*42*) instead dropped the fully connected layers and instead added three locally connected spatialized layers, with coupled excitatory and inhibitory processes. When trained on faces, objects, and scenes, these layers show increasing clustering to these categories. In both approaches, topographic constraints are directly integrated into the feature learning process.

In contrast, we cast the problem of topography as one of data-manifold mapping, which is more closely related to the approaches taken by Keller *et al*. (*44*) and Zhang *et al*. (*43*). Keller *et al*. (*44*) trained a topographic variational autoencoder which, like our SOM, was also trained on from the features of a pretrained AlexNet model (though appended after the final convolutional stage). This topographic layer is also a grid of units (though, with a circular topology), initialized into the deep net feature space, and trained to maximize the data likelihood using an algorithm related to independent component analysis. Similarly, Zhang *et al.* (*43*) leveraged a pretrained AlexNet (though, they used the final output layer, first reducing it to four dimensions using principal components analysis), and then trained a SOM followed by an additional warping step to map the SOM onto the ventral OTC. Both these approaches probe the resulting tuned map with some of the same stimulus sets as in the present work, though we all used different analysis methods to compute activations and to quantify the spatial organization, resulting in some differences [e.g., both Keller *et al.* (*44*) and Zhang *et al.* (*43*) report the presence of body-selective regions]. As a whole, these methods use a topographic layer to reveal the untangled data manifold of a pretrained feature space, rather than to constrain the learning of the features themselves.

Currently, a deeper theoretical understanding is needed to relate these emerging DNN-topographic approaches to each other and to prior approaches of modeling cortical topography (*85*–*88*). Given our formulation of topography, we do not take the present model as a mechanistic model of cortical topographic development, but rather as one that captures a rather normative account (*46*, *50*). For a more mechanistic model of cortical topography, we see the relevant level of abstraction as one that takes on the full topographic challenge, learning the growth rules to connect a grid of units into a useful hierarchical network architecture [likely leaning on an eccentricity-based scaffold and the activity of retinal waves to initialize the architecture (*89*–*92*)]. However, many other approaches are also possible which reflect different abstractions, e.g., incorporating differentiable SOM stages after each hierarchical layer block. Broadly speaking, there is clear theoretical work to do integrating the goal of a smoothly mapped data manifold with the learning processes that yield structured connections, complex feature tuning, and hierarchical untangling of the input as accomplished by DNNs.

Finally, complementing these computational approaches, there is a clear need to develop quantitative metrics for comparing topographic activation similarity, which takes into account distance on a cortical sheet (e.g., Wasserstein distance). Recent open, large-scale condition-rich fMRI datasets are now available [e.g., NSD dataset (*93*) and THINGS dataset (*94*)], which can enable the development of cortical topographic metrics beyond these macro- and mesoscale signatures probed for here. Thus, going forward, there is clear work to do toward mapping these computational models more directly to the cortex and assessing how they succeed and fail at capturing the systematic response structure to thousands of natural images across the cortical surface.

## MATERIALS AND METHODS

### Spatializing the representational space of a deep net with a self-organizing map

#### Input data and SOM parameters

We applied a Kohonen SOM algorithm (*45*) to the multidimensional feature space of the relu7 stage of a pretrained AlexNet (*52*) sourced from the Torchvision (PyTorch) model zoo (*95*). The input data are a set of *p* points encoded along *f* feature dimensions. Here, the *p* points reflect the 50,000 images from the ImageNet validation set, and the *f* dimensions reflect the 4096 features from the relu7 stage of the network, i.e., *f* ∈ {*f*_1_,  *f*_2_, …., *f*_4096_}. In addition, we specify the number of SOM units (here, 400 units) as an input parameter and set additional training hyperparameters related to the number of training epochs, and how the learning rate and map neighborhood influence changes over the course of map training, detailed below.

#### SOM training

The first stage of the algorithm is to define the map shape, and then initialize the tuning for each unit on the map such that the map spans the first two principal components of the input data. Computing the principal components over 50,000 points in the 4096-D input space is computationally intensive; thus, we created a smaller sample of 400 images over which we computed the top two eigenvectors and eigenvalues. In a control analysis, we varied the images and the size of this subset over which the principal components were calculated and found that this choice had negligible impact on the final results (see fig. S10).

The first step is to determine the aspect ratio of the SOM, based on the ratio of the top two eigenvalues. In the case of the relu7 feature space, the aspect ratio of the data was ~1; thus, the input parameter of 400 map units lead to the construction of a 20 × 20 (*W ×H*) map grid. Next, each unit in the 20 × 20 grid is placed in the 4096-D space such that the entire map is centered along the plane formed by the first two eigenvectors, scaled by their respective eigenvalues (see the top row of fig. S1). To scale the eigenvectors, we compute unit vectors along the two principal components and multiply them with the square root of their corresponding eigenvalues. Here, we refer to the location of a map unit in the 4096-D space as that unit’s tuning vector and the set of all map tuning vectors as the codebook, which is of size *W* × *H* × *f*, here 20 × 20 × 4096. This method of initialization ensures that the map is matched to the relative contributions of the top two major dimensions/axes of variation in the input data and allows for a more consistent embedding in this high-dimensional input space.

After initializing the map tuning vectors, the next stage is to fine-tune and iteratively update these tuning vectors to better capture the input data manifold. All 50,000 images from the ImageNet validation set were used during fine-tuning. The full image set is seen every epoch and the SOM was tuned for a total of 100 epochs. Within each epoch, the map tuning updates operate over a smaller batch of images. Our batch size was 32 images. For every image in the batch, we first identify the single SOM unit whose 4096-D tuning vector is closest to that image’s 4096-D embedding in the DNN feature space, using the Euclidean distance metric. This SOM unit becomes the image’s “best matching unit” or BMU (see Eq. 1).

$$\mathrm{BMU}={\mathrm{argmin}}_{w,h}\sqrt{\sum_{f=0}^{f=4096}{\left[{input}_{f}-{\mathrm{tuning}}_{(w,h),f}\right]}^{2}}$$ (1)

Here *input*_*f*_ is the image’s DNN activation value on the *f*th feature dimension and tuning_(*w*,*h*),*f*_ is the scalar value, for the *f*th feature dimension, on the tuning vector of a map unit that is situated in the *w*th row and *h*th column of the SOM grid. Hence, the BMU is the SOM unit with the minimum Euclidean distance to the image’s feature vector (i.e., *input*) among all the SOM units. Next, for each of the BMUs (32 per batch), we adjust its tuning vector and the tuning vectors of other map units that are within a neighborhood of the BMU such that they are closer to the 4096-D location of the corresponding image. This updated rule, at a particular time step *t* (i.e., epoch), is formulated in Eq. 2.

$${tuning}_{t+1}={tuning}_{t}+L_{t}\eta_{t}(input-{tuning}_{t})$$ (2)

Here the *tuning* vector of each map unit is adjusted toward the *input* based on the learning rate function *L_t_*, and the neighborhood function η*_t_*. The learning rate (*L_t_*) controls the magnitude of the tuning adjustment, which slowly decays to make smaller adjustments over time, following Eq. 3. The initial learning rate *L*_0_ was set at 0.3 and *T* denotes the total number of epochs (set to 100).

$$L_{t}=L_{0}\left(1-\frac{t}{T}\right)$$ (3)

The neighborhood function η*_t_* measures the influence a map unit’s distance from the BMU has on that map unit’s learning. Intuitively, units that are closer to the BMU need to be updated more strongly as compared to units that are further away. This is expressed using a Gaussian widow (see Eq. 4) that is centered on the computed BMU with a radius/standard deviation of σ*_t_*.

To center the window on the BMU, Eq. 5 is used which computes the L2-distance between a unit present in the *i*th row and *j*th column and a BMU that is situated in the *w*th row and *h*th column of the SOM grid. Note that this distance is computed directly on the 2D SOM grid and not in the 4096-D input space. This constraint generally encourages neighboring units on the map to encode nearby parts of the high-dimensional input space. For the radius of the neighborhood window, we start with a radius of σ*_o_* that covers approximately half of the map (hence for the map of shape 20*20, it was set at **10**). This radius exponentially decays over the training epochs following Eq. 6. By starting with a larger neighborhood and gradually shrinking the neighborhood influence, the map is less influenced by image order and batch size and stabilizes in a smoother larger-scale embedding

$$\eta_{t}=e^{\frac{-{D_{\mathrm{map}}}^{2}}{2{\sigma^{2}}_{t}}}$$ (4)

where

$$D_{\mathrm{map}}({\mathrm{BMU}}_{w,h},{\mathrm{unit}}_{i,j})=\sqrt{(w-i{)}^{2}-{\left(h-j\right)}^{2}}$$ (5)

$$\sigma_{t}=\sigma_{o}\left(1-\frac{t}{T}\right)$$ (6)

Map-tuning updates are made for each batch, with a single epoch completed after all 50,000 images have been presented. At the next epoch (i.e., next time step *t*), the learning rate and neighborhood parameters are updated (using Eqs. 5 and 6) and the process is repeated, continuing for a total of 100 epochs. Because of the decay of the learning rate, the training stabilizes at the end of the total epochs, and we do not find large differences in the codebook with more training epochs.

A standard measure of map fit to the input data is the quantization error (QE), which is the average Euclidean distance between the input image’s DNN features and the tuning of their corresponding computed BMUs. As the map is fine-tuned, this tuning better matches the input data, and the QE decreases. A plot of the QE over epochs is shown in fig. S2A. In fig. S2 (B and C), we visualize the pairwise tuning of SOM units as a function of their distance on the 2D grid. The tuning similarity reduces as distance on the 2D grid increases as expected via the constraint introduced in Eq. 4.

At the end of the fine-tuning phase, we have a trained SOM, or “simulated cortex”—a grid of units of shape 20 × 20, each tuned systematically in the high-dimensional space (ℝ^4096^) to encode the data manifold of the input of natural images in the relu7 feature space of AlexNet.

### Simulated cortical activations

To get the activations of new images on the simulated cortex, we pass the image through the pretrained AlexNet and compute its 4096-D features in the *relu7* space (i.e., *input* vector for that image). Each unit on the SOM also has an associated *tuning* vector in this feature space (ℝ^4096^) and can be conceived of as a filter, i.e., a weighted combination of the DNN features. Thus, we compute the activation of each SOM unit by taking the dot product of that unit’s tuning vector and the image’s relu7 features using Eq. 7. Across all map units, this creates a spatial activation profile for the image.

$$\mathrm{Activation}=\sum_{f=0}^{f=4096}{tuning}_{f}\;*\;{input}_{f}$$ (7)

### Stimulus sets

The following stimulus sets were used to probe the spatial topography of the SOM: (i) Konkle and Caramazza (*9*): Animacy × Size images – 240 color images of big animals, small animals, big objects, and small objects (60 each); (ii) Long *et al.* (*11*): Original and Texform Animacy × Size images – 120 grayscaled luminance matched images and 120 corresponding texform images, depicting animals and objects of big and small sizes (30 each); (iii) Cohen *et al*. (*59*): Category-localizer stimulus set 1: 240 total grayscaled luminance-matched images of faces, bodies, cats, cars, hammers, phones, chairs, and buildings (30 each); (iv) Konkle and Caramazza (*9*): Category-localizer stimulus set 2: 400 total color images of faces, bodies, scenes, objects, and block-scrambled objects on a white background (80 each).

### Preference maps

Preference maps were created following the same procedures as used in fMRI analysis (*9*). Simulated cortical activations (Eq. 7) were computed for all individual images from the stimulus set. For each map unit, we computed the average activation for each targeted image condition (e.g., averaging across all animal images or all object images). Next, we identify the “preferred” condition, eliciting the highest average activation, and calculated this response preference. For two-way preference maps, the preference strength is the absolute difference between the mean activations of the two categories. For *n*-way contrasts, the preference strength is the absolute difference between the activation of the preferred condition and the second-most activation condition. We visualize the response preferences using custom color maps that interpolate between gray and the target color for each condition, where the color of each unit reflects the preferred category color, and the strength of the preference scales the saturation. The mapping between the color palette and the data values is controlled with color limit parameters and was matched across the multiple color maps in the preference map visualization.

### Category selectivity metrics

To compute maps of category selectivity, we used the following procedure. First, we computed the simulated cortical activations (using Eq. 7) for all images in the localizer set. Next, for each unit on the map, we computed the mean and variance of activation responses for images from the target category (i.e., ${\bar{X}}_{\mathrm{target},}\;\sigma_{\mathrm{target}}^{2}$) and for all remaining images (i.e., nontarget condition; ${\bar{X}}_{\mathrm{remaining},}\;\sigma_{\mathrm{remaining}}^{2}$) and computed d′ following Eq. 8

$$d^{\prime }=\frac{{\bar{X}}_{\mathrm{target}}-{\bar{X}}_{\mathrm{remaining}}}{\sqrt{\frac{\sigma_{\mathrm{target}}^{2}+\;\sigma_{\mathrm{remaining}}^{2}}{2}}}$$ (8)

For robustness, we additionally computed another standard measure—*SI* for each map unit, which differs slightly from d′ in how it is normalized (i.e., by the means, rather than the variances), following Eq. 9. Both metrics yielded convergent results (see fig. S5)

$$SI=\frac{{\bar{X}}_{\mathrm{target}}-{\bar{X}}_{\mathrm{remaining}}}{{\bar{X}}_{\mathrm{target}}+{\bar{X}}_{\mathrm{remaining}}}$$ (9)

For each map, we also computed a nonuniformity score, based on how different the selectivity map was from a uniform distribution. For each selectivity map, we normalize the d′ scores using a softmax function to get a probability distribution *P* of the selectivity on the map. We then compare this to a completely uniform distribution of selectivity *Q*, using the Jenson Shannon distance following Eq. 10, where *KL*(*P*‖*Q*) is the KL divergence between distribution *P *and *Q*

$$\text{JS Distance}=\sqrt{\left[\frac{KL(P\|Q)}{2}+\frac{KL(Q\|P)}{2}\right]}$$ (10)

### Comparing selectivity maps and preference maps

To quantify the relationship between category-selective maps and the animate-inanimate preference maps, we used an ROC analysis, following the procedures used in (*9*). The procedure is, as follows, described here for the specific case of comparing the map of face-d′ and the map of responses of animate-inanimate preferences. First, the face d′ values are sorted across all 400 map units on the 20∗20 grid. For each step in the analysis, the topmost selective units are selected, starting from the top 1% most face-selective, and then the top 2% most face-selective, and so on, until we consider all 100% of the map units. For each step, we separately compute the proportion of all animate-preferring SOM units and the proportion of all inanimate-preferring units that overlap with these face-selective units. Across all steps of the analysis, as an increasing number of units from the face selectivity map are considered, the procedure sweeps out an ROC curve between (0,0) and (1,1). For example, if all of the topmost face-selective units were also all animate-preferring units, then this curve would rise sharply (indicating rapid filling of the animate-preferring zone), before leveling off. Thus, the area between the curve and the diagonal of this plot (AUC) was used as a threshold-free measure of overlap between face selectivity and the animacy organization. We computed these ROC curves for both the face- and scene-selective contrasts, computed over both localizer sets.

To measure the significance of this relationship between category selectivity and the large-scale preference organization, we used permutation tests, i.e., we iterated through 1000 simulations, and, for each simulation, we randomly shuffled the selectivity measure estimates. For each shuffled simulation, we plot the ROC curve across the thresholds and evaluate the AUC measure. The proportion of these simulated AUCs that are higher than the originally measured (unshuffled) AUC gives us the significance of the measured AUC overlap.

### Representational geometry and multidimensional scaling plots

The 240 images of animals and objects of different sizes were passed through the pretrained AlexNet, and the 4096-D features in the *relu7* space were extracted for each image. Next, pairwise correlations were conducted over these features using the 1-Pearson correlation measure, yielding a representational dissimilarity matrix of 240 × 240 matrix. This matrix was inputted into a standard multidimensional scaling (MDS) algorithm with output dimensionality set to 2. Images that are more similarly represented in the DNN feature space are closer to each other in the 2D MDS plot.

### Gradient-based image synthesis

Given that the tuning of each unit on the SOM can be conceived of as a weighted combination of the relu7 features, we can conceptualize a SOM as an additional fully connected layer on the top of the relu7 layer with a weight matrix of shape 4096 × 400, i.e., the 4096-D tuning vector for each of the 400 units on the 20 × 20 grid of the SOM. This model (i.e., DNN + attached layer from SOM tunings) is end-to-end differentiable with respect to the input images. As a result, we can start with a noise image and iteratively update it using gradient ascent such that the optimized image increases the output for a selected output unit (which is equivalent to increasing the simulated cortical activation of a unit on the SOM). We use the torch lucent library (https://github.com/greentfrapp/lucent) to synthesize these images.

## Supplementary Materials

### This PDF file includes:

Figs. S1 to S13 Analysis (Pixel representation)

## Notes

View/request a protocol for this paper from *Bio-protocol*.
