# Supplementary material for: Cortical topographic motifs emerge in a self-organized map of object space
Source: Sci Adv. 2023 Jun 21;9(25):eade8187. doi: 10.1126/sciadv.ade8187 (PMC10284546; doi:10.1126/sciadv.ade8187)
Supplement: Supplementary file 1 — Figs. S1 to S13 Analysis (Pixel representation) [file sciadv.ade8187_sm.pdf]

Science Advances
AAAS

Supplementary Materials for

Cortical topographic motifs emerge in a self-organized map of object space

Fenil R. Doshi and Talia Konkle

Corresponding author: Fenil R. Doshi, fenil_doshi@fas.harvard.edu

Sci. Adv. **9**, eade8187 (2023)
DOI: 10.1126/sciadv.ade8187

This PDF file includes:

Figs. S1 to S13
Analysis (Pixel representation)

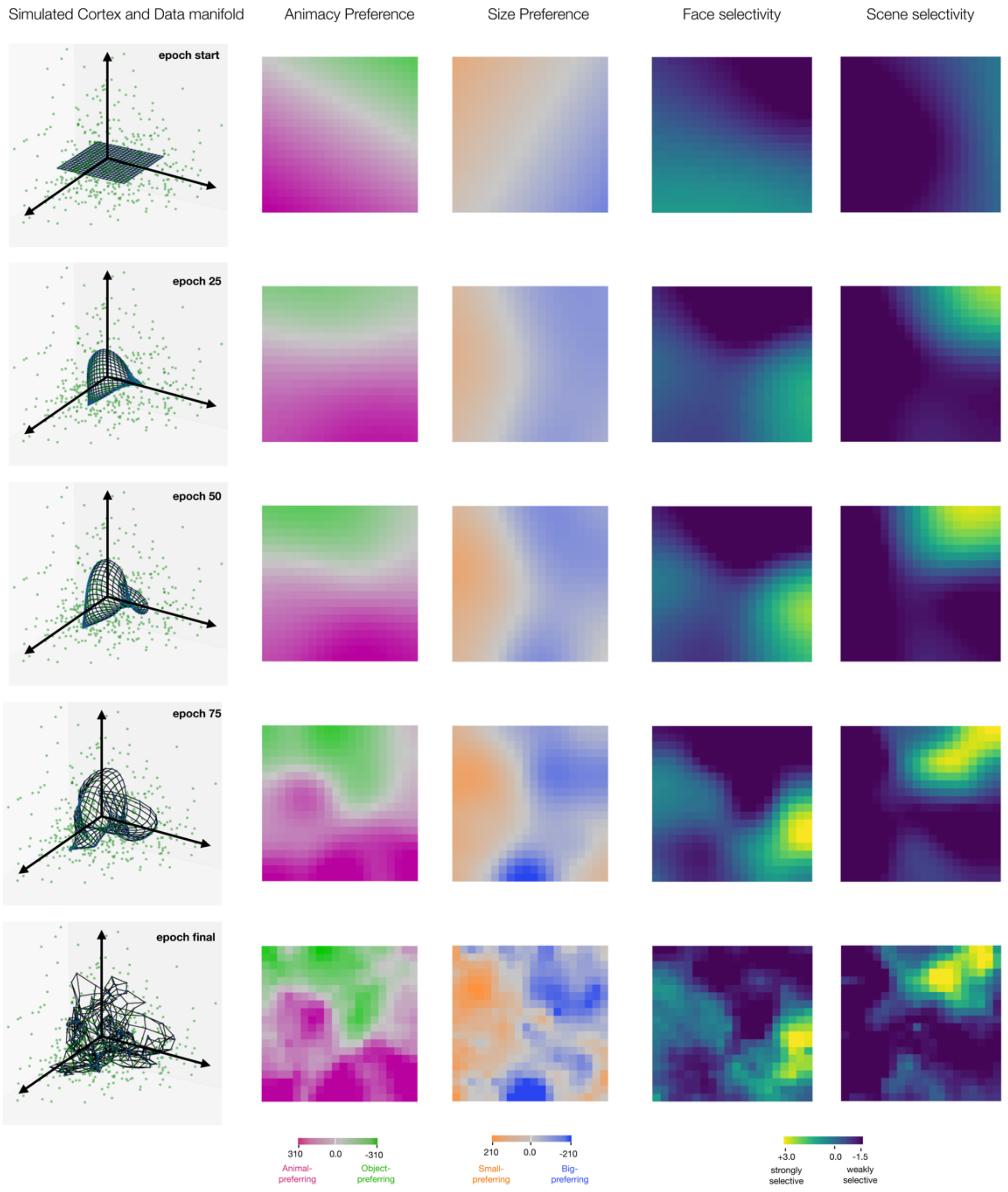

Fig. S1. SOM training over time.

Training (Initialization and Fine-tuning) stages of the SOM. In each row we visualize the simulated cortex in context of the input data's PC-space – the green points depict the location of images in the input feature space (dnn features) and the black connected points depict the tuning of SOM map units in this PC-space. We also visualize the animacy and size preference, and face- and scene-selectivity on this simulated cortex for every stage in the training process.

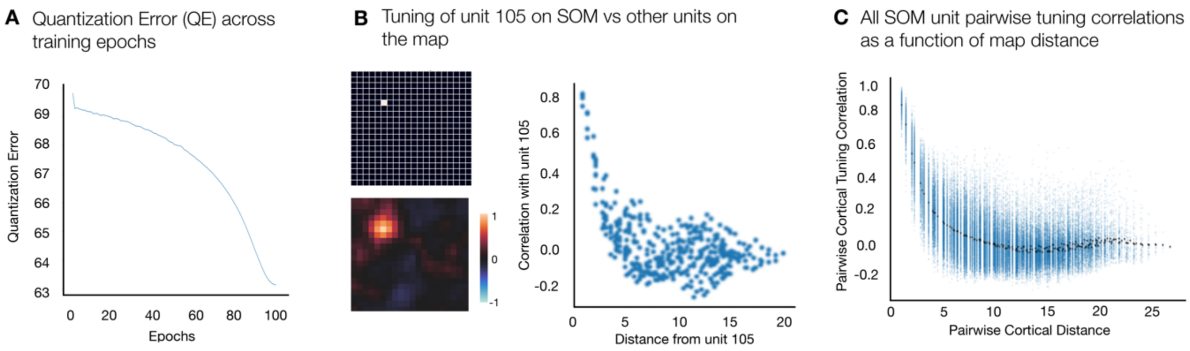

Fig. S2. SOM Quality Metrics.
(A) Quantization error of the SOM as a function of training epochs (B) Pairwise tuning similarity between one example SOM unit with all other units on the SOM, plotted as a heat map (top) and a scatter plot (below), with tuning similarity (correlation) along the y-axis, plotted as a function of map distance between units (euclidean) on the x-axis (C) Scatter plot with tuning similarity between every pair of units on the SOM on the y-axis and the map distance (euclidean) between pair of units on the x-axis.

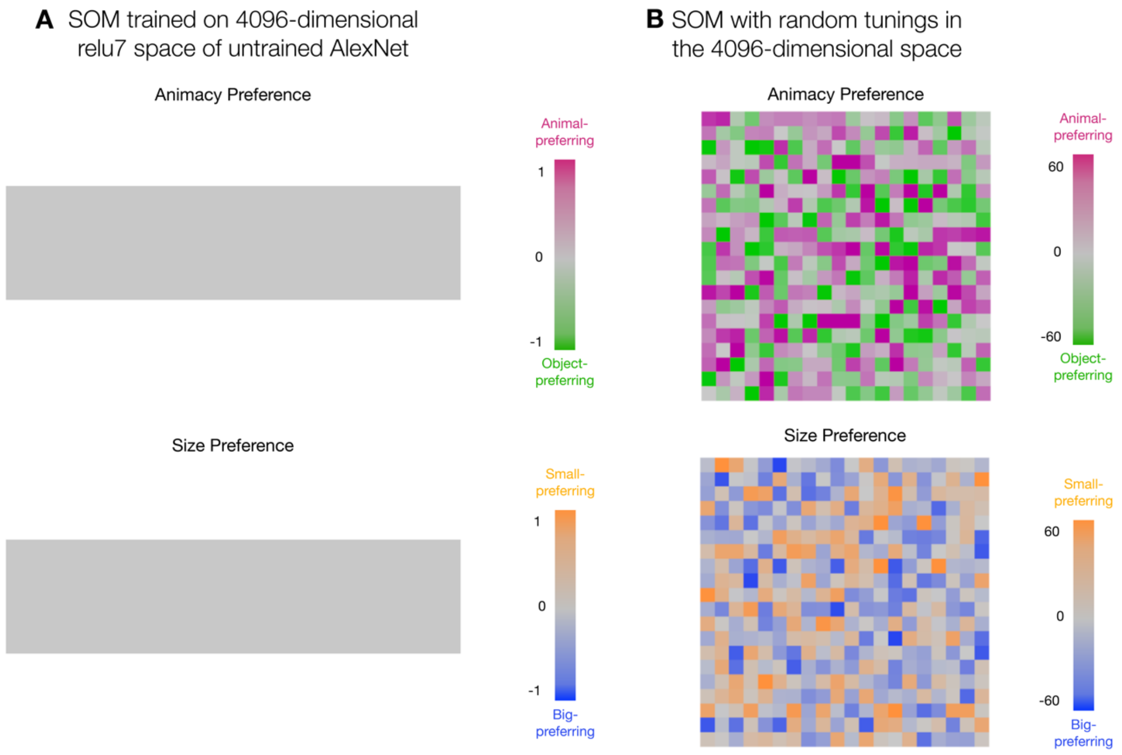

Fig. S3. Control SOMs.
(A) *A new SOM was fit into the relu7 (*$\mathbb{R}^{4096}$*) feature space of an untrained Alexnet which resulted in an 9x38 SOM. Animacy (animals vs objects) and Size (big vs small entities) preference on this map. (B) Animacy and size preference on a 20*20 SOM when the map is randomly tuned in the 4096-dimensional relu7 space of a trained Alexnet.*

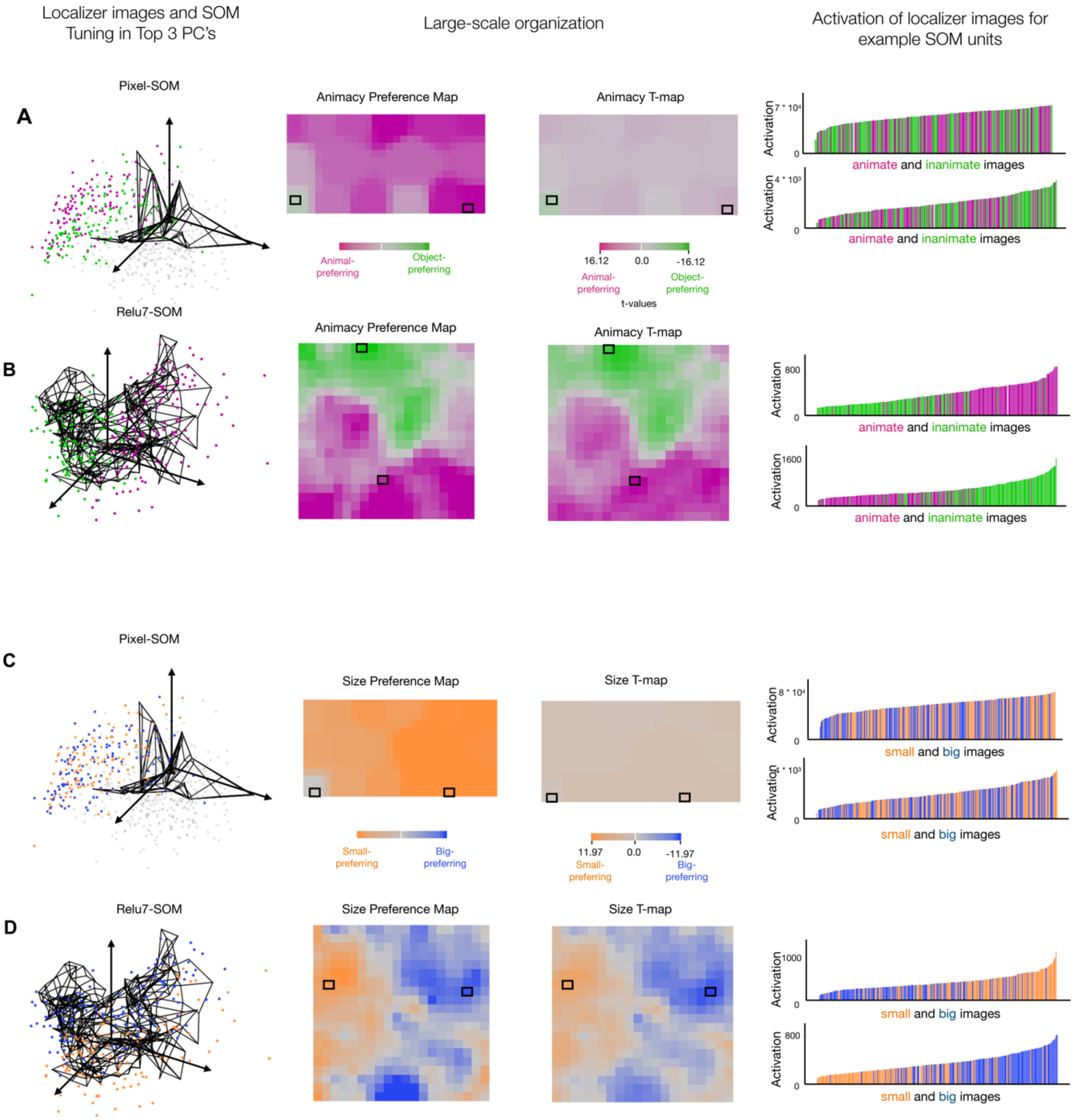

Fig. S4. Pixel-based vs Relu7 SOM comparisons.
(A) First subplot: A plot of the first 3 principal components of the pixel-space representation. Colored dots reflect the images: purple dots reflect animate images; green dots reflect inanimate images; gray dots reflect a sample of 400 ImageNet images. The black grid is the SOM, projected into the pixel-space. Second subplot: Preference map showing the difference in average activation for animate and inanimate images for each SOM unit. Third subplot: T-map visualize the separability of animate vs inanimate images for each SOM unit. Fourth subplot: Two units were

selected from the t-map, which show the maximum t-values for an animate preference or inanimate. For both of these units, the degree of activation (y-axis) is plotted for all 240 localizer images (x-axis), sorted by their activation. Animate images are colored with purple lines, and inanimate images are colored with green lines. The top plot shows the unit with the strongest animacy preference, the bottom plot show the unit with the strongest inanimate object preference. (B) The same four subplots are show, here for the Relu7 feature space of a pre-trained Alexnet. A clearer distinction between animate and inanimate images is seen on the preference and the t-maps. (C) The same four subplots are shown, here for the pixel-space representation, considering the real-world size distinction between images of big and small entities. Across all plots, orange reflects images of small entities, while blue reflects images of big entities. (D) The same four subplots are shown, here for the Relu7 feature space of a pre-trained Alexnet, now focusing on the real-world size distinction. A clearer distinction between big and small images is seen on the preference and the t-maps in this trained feature space. See supplementary analysis section for details and discussions of these results.

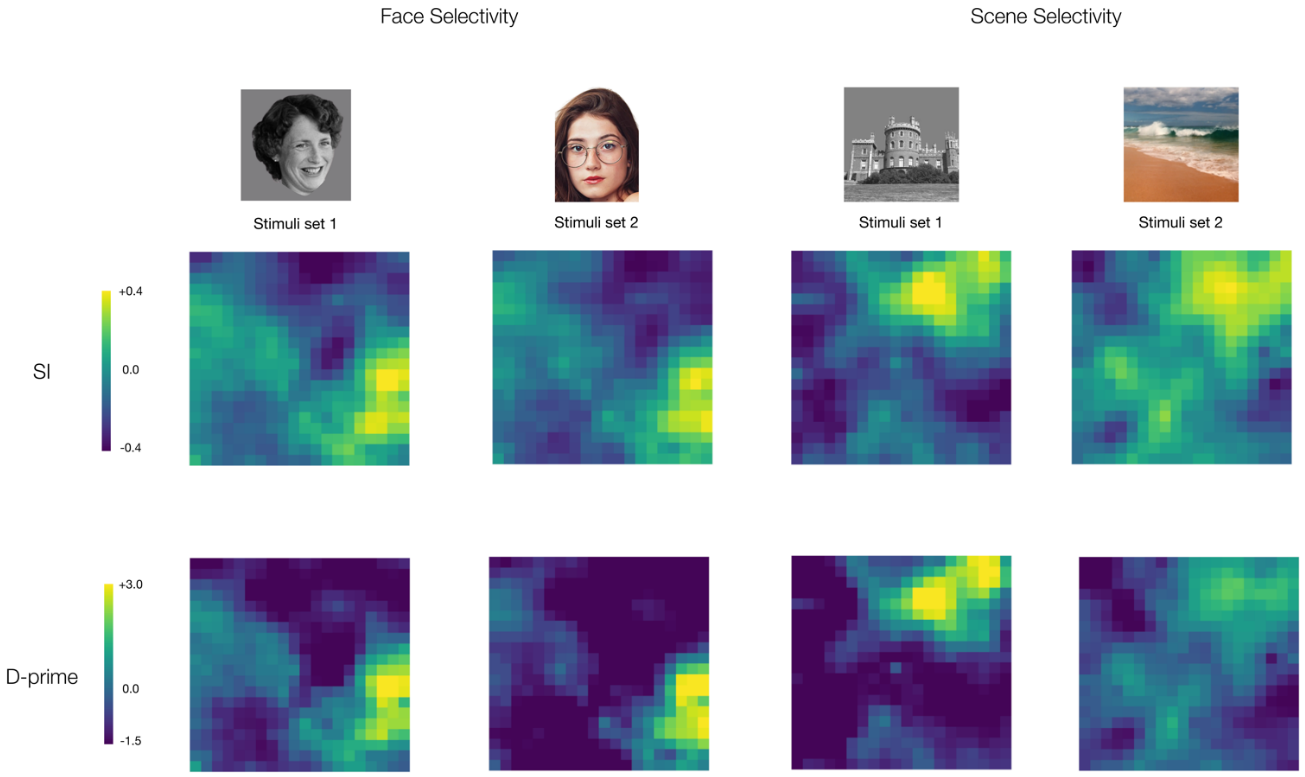

Fig. S5. Face and Place Selectivity with D' and Selectivity Index comparisons.
Category selectivity for faces and scenes within the two stimuli sets, measured using Selectivity Index (SI) and D-prime measure.

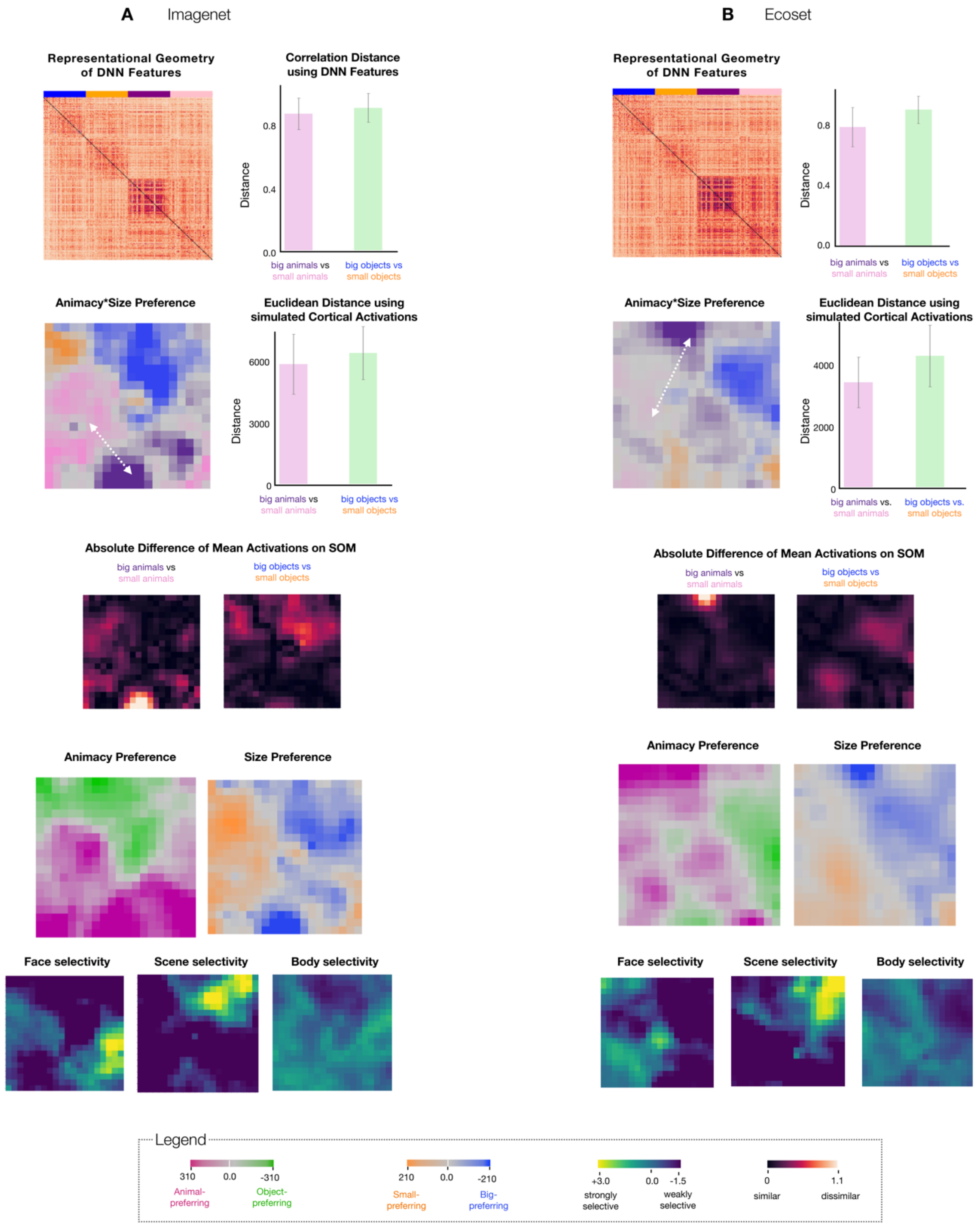

Fig. S6. SOMs trained on different visual diets.

Comparing the large-scale organization between models (deepnet and SOM) trained on different visual diets – imagenet vs ecoset. For each visual experience, we visualize: (i) Representational Geometry of images (stimuli from (9)) in the deep net feature space. (Left) RDMs based on correlational distance. (Right) Bar plots showing the image-level pairwise correlational distance

between dnn features of big and small animals, and dnn features of big and small objects. (ii) (Left) 4-way preference map on the simulated cortex among big objects, small objects, big animals, and small animals and (Right) Bar plots showing the image-level pairwise euclidean distance between simulated cortical activations of big and small animals, and simulated cortical activations of big and small objects using the same stimuli as in (i). (iii) Heatmaps showing the absolute difference of mean simulated cortical activations based on size for animals (i.e. big animals vs. small animals) and objects (i.e. big objects vs. small objects) (iv) Animacy (animals vs. objects) and Size (small vs. big) preferences on the simulated cortex. (v) Face-, Scene-, and Body-selectivity on the simulated cortex measured using the d-prime measure. Stimulus from (59) were used to compute the selectivity maps.

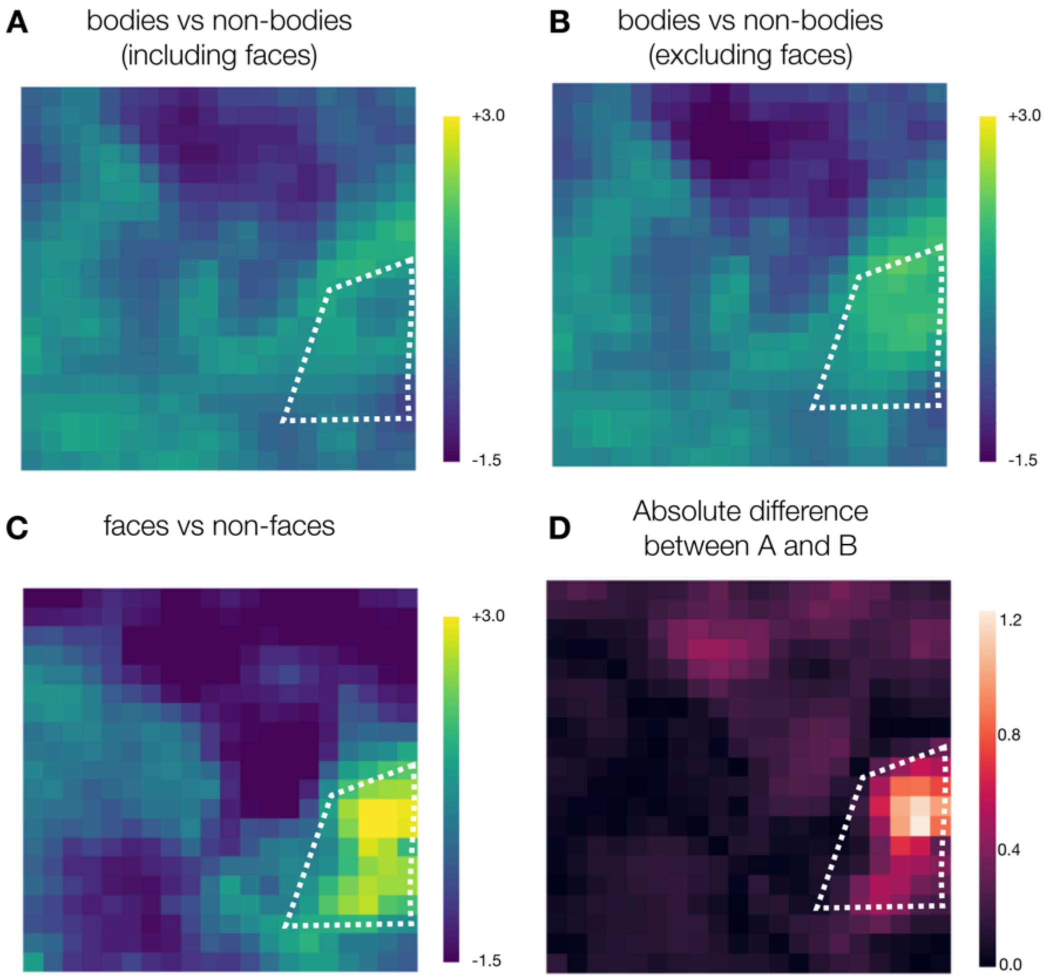

Fig. S7. Body selectivity with faces included and excluded.
(A and B) Body-selectivity on the simulated cortex with faces included and excluded while computing the d-prime selectivity map. (C) Face-selectivity on the simulated cortex (D) Mean Absolute difference between (A) and (B). The white lines demarcate the most face-selective zone.

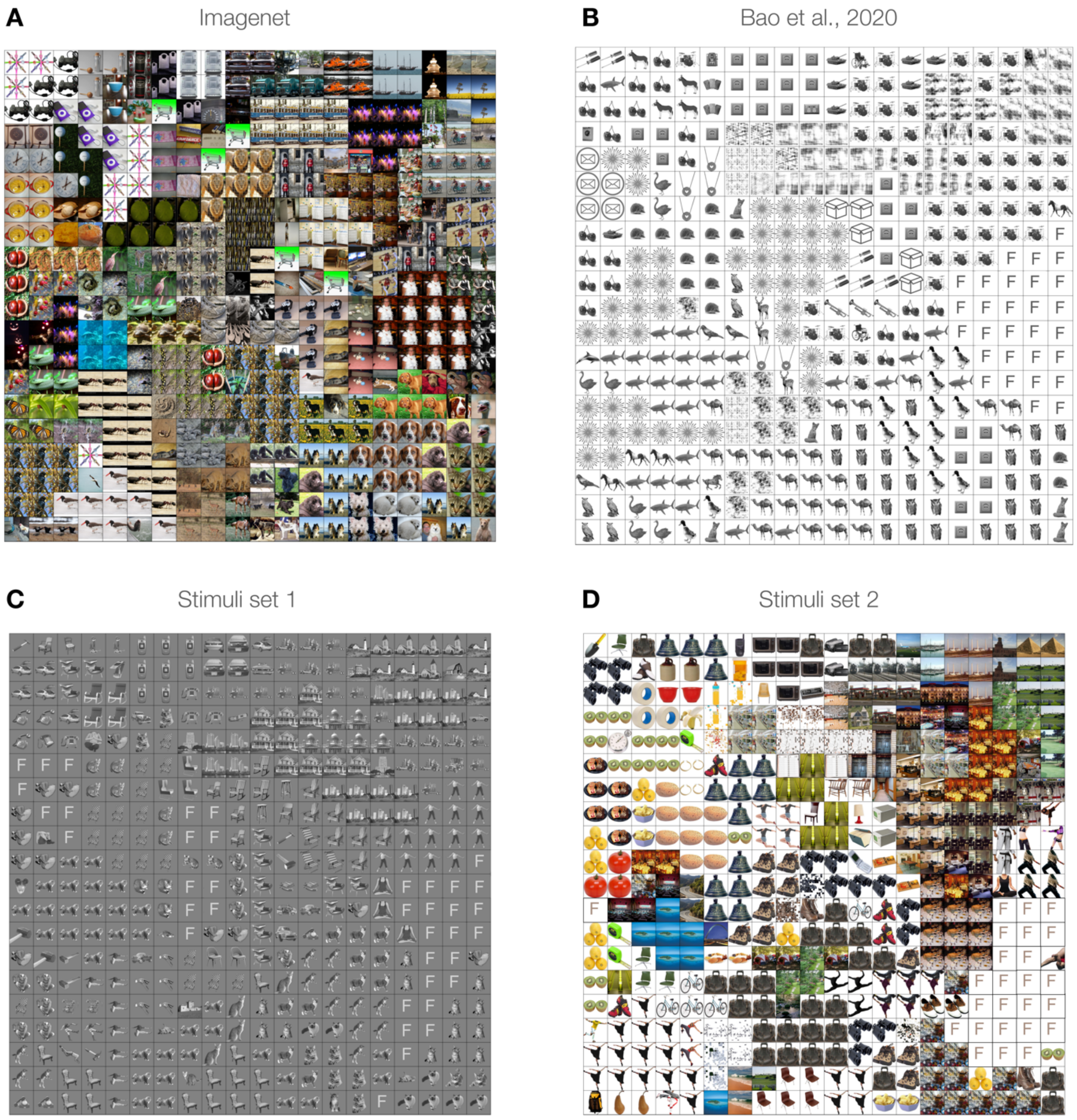

Fig. S8. Maximally activating images.

*Map of images that maximally drive map units on the 20*20 grid computed over different stimulus sets: (A) ImageNet validation set (B) stimuli used in Bao et al., 2020 (26) (C,D) two localizer sets used in this study. Face images replaced with letter F.*

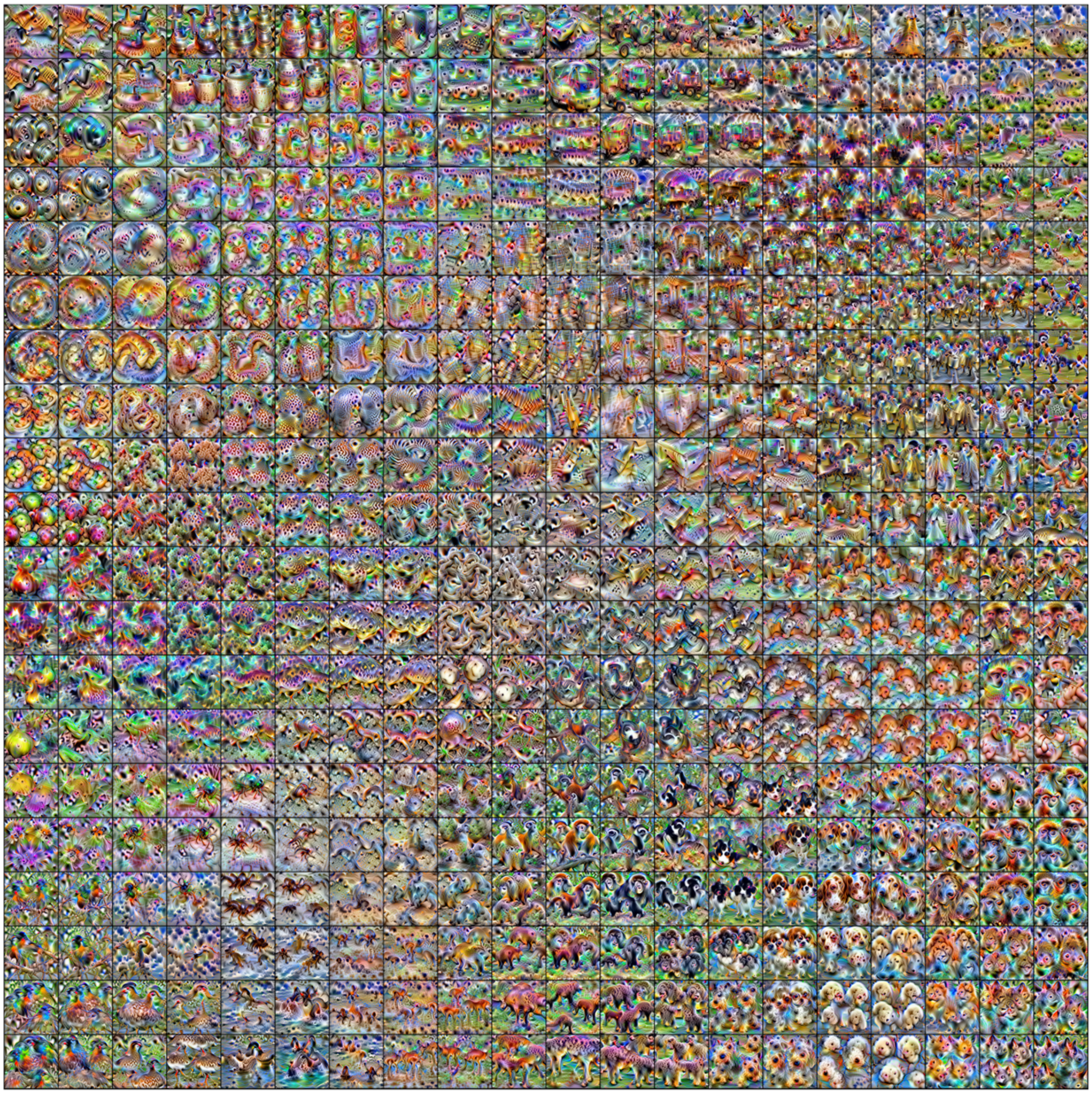

Fig. S9. Maximally activating synthetic images.
*Synthesized maximally activating images, generated using gradient ascent, for all map units on the 20*20 grid.*

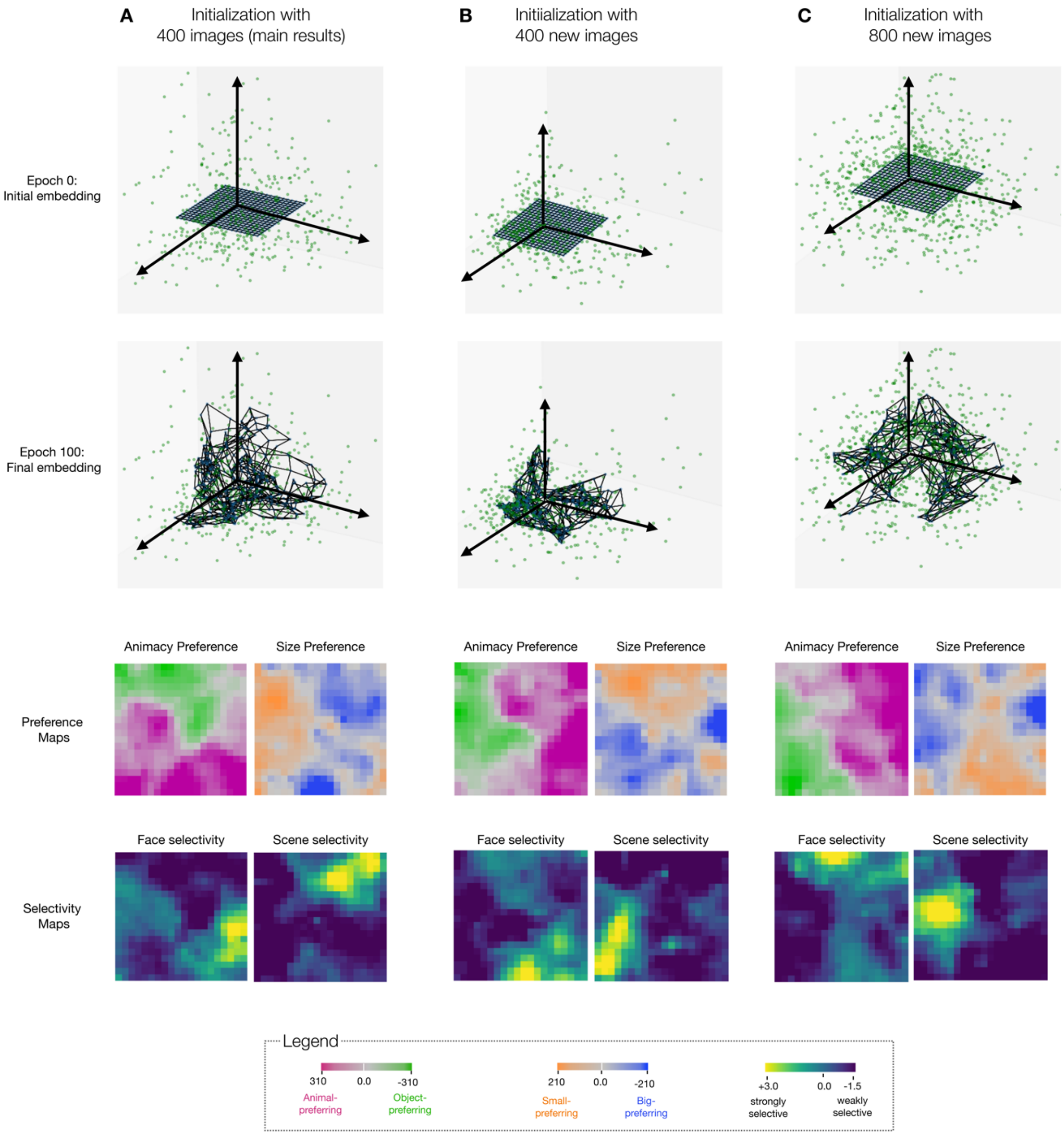

Fig. S10. Varying images used to initialize SOM.

Each column is a trained map initialized with different images from the SRS/Imagenet validation set. First column uses 400 images (same as the ones reported in the main figures), the second column uses 400 different images, and the third column uses 800 images. The training images remain the same for all 3 variations. Top and Second row: Visualization of the map units projected into the first three principal components of the input space before (epoch 0) and after training (epoch 100). Third row: Large-scale organization of animacy and size after training. Bottom row: Category selectivity for faces and scenes after training.

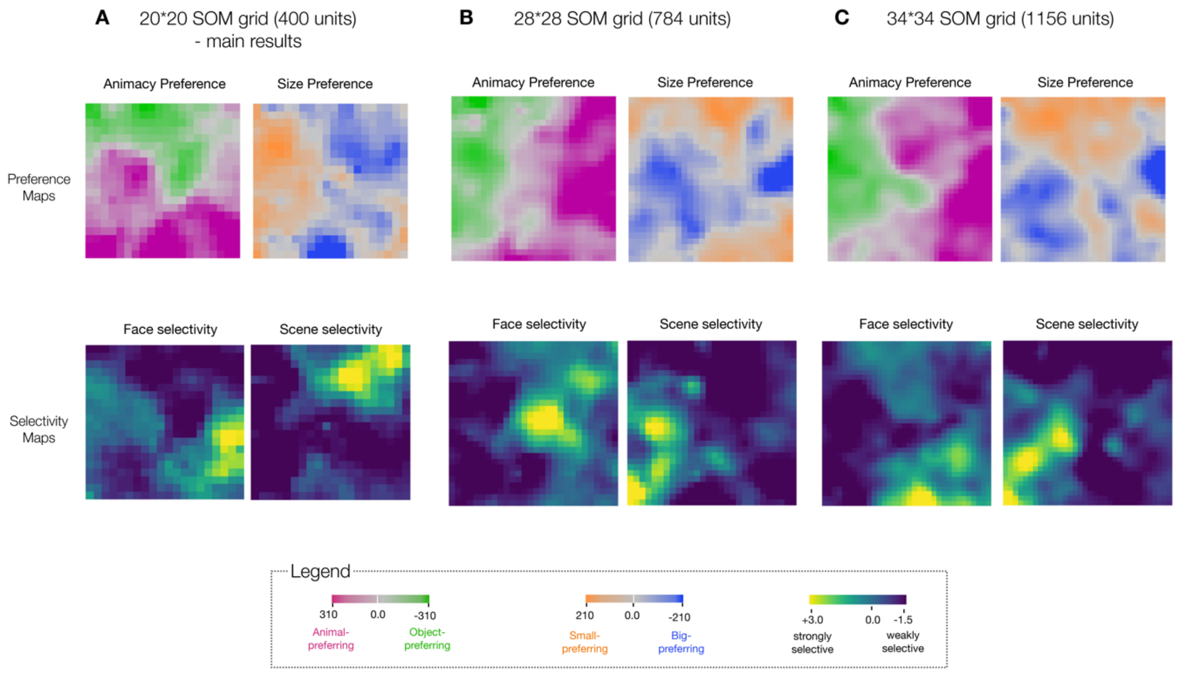

Fig. S11. Varying the SOM size.
Each column is a trained map. The size of the map varies from small to large, from left to right. Top row: Large-scale organization of animacy and size. Bottom row: Category selectivity for faces and scenes.

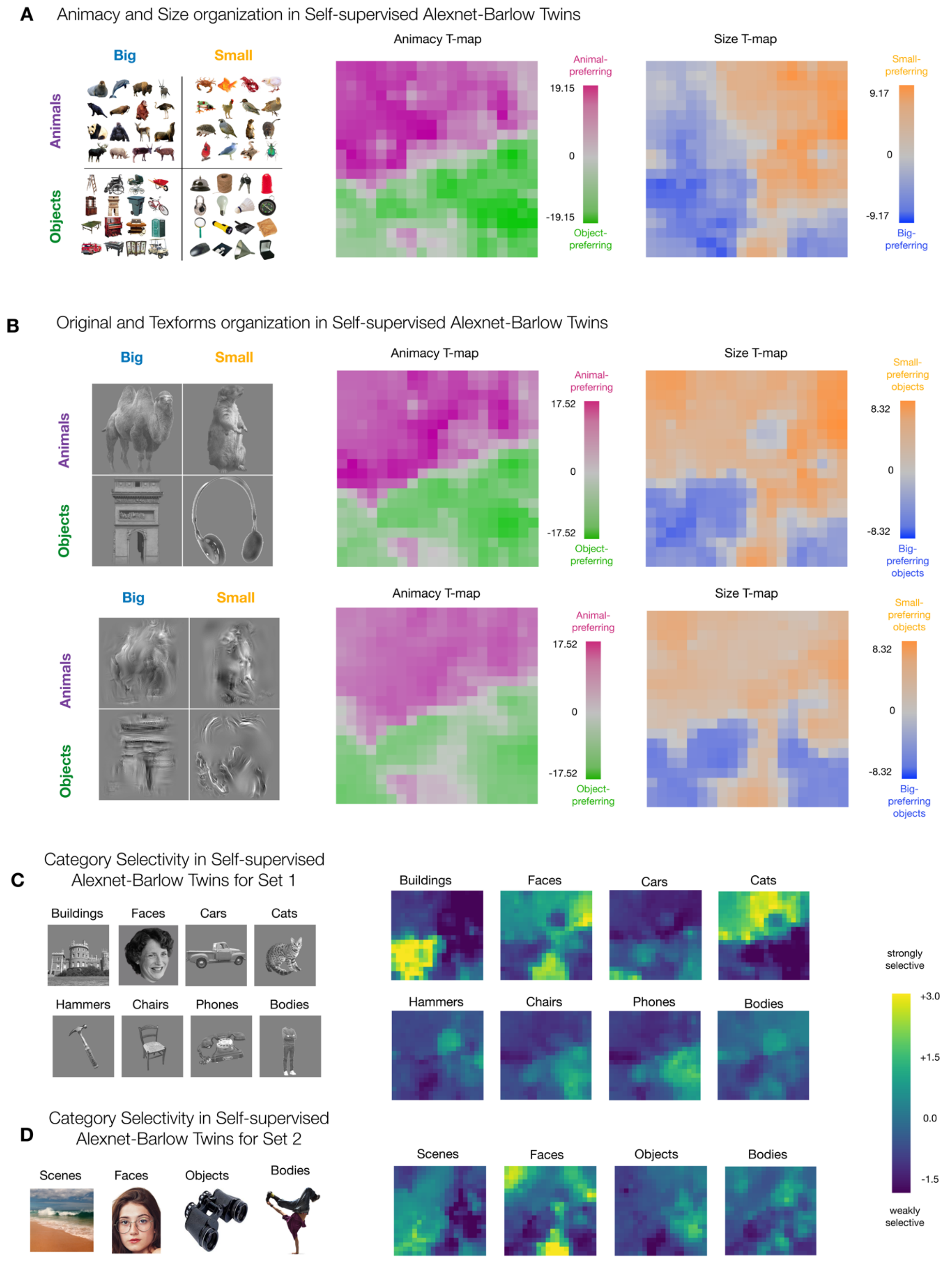

Fig. S12. Topographic motifs in a self-supervised DNN (Barlow Twins).
(A) Animacy and size distinctions on the SOM for images containing animals and objects of different sizes (left). Each unit on the SOM is colored by its response preference (t-statistic) to either animal

or object images (center) and to images of either big or small entities (right). (B) The same three subplots, here shown for grayscaled original and texform images. (C, D) Example images from each category in Set 1and 2 and the corresponding category-selective maps computed using the d-prime metric.

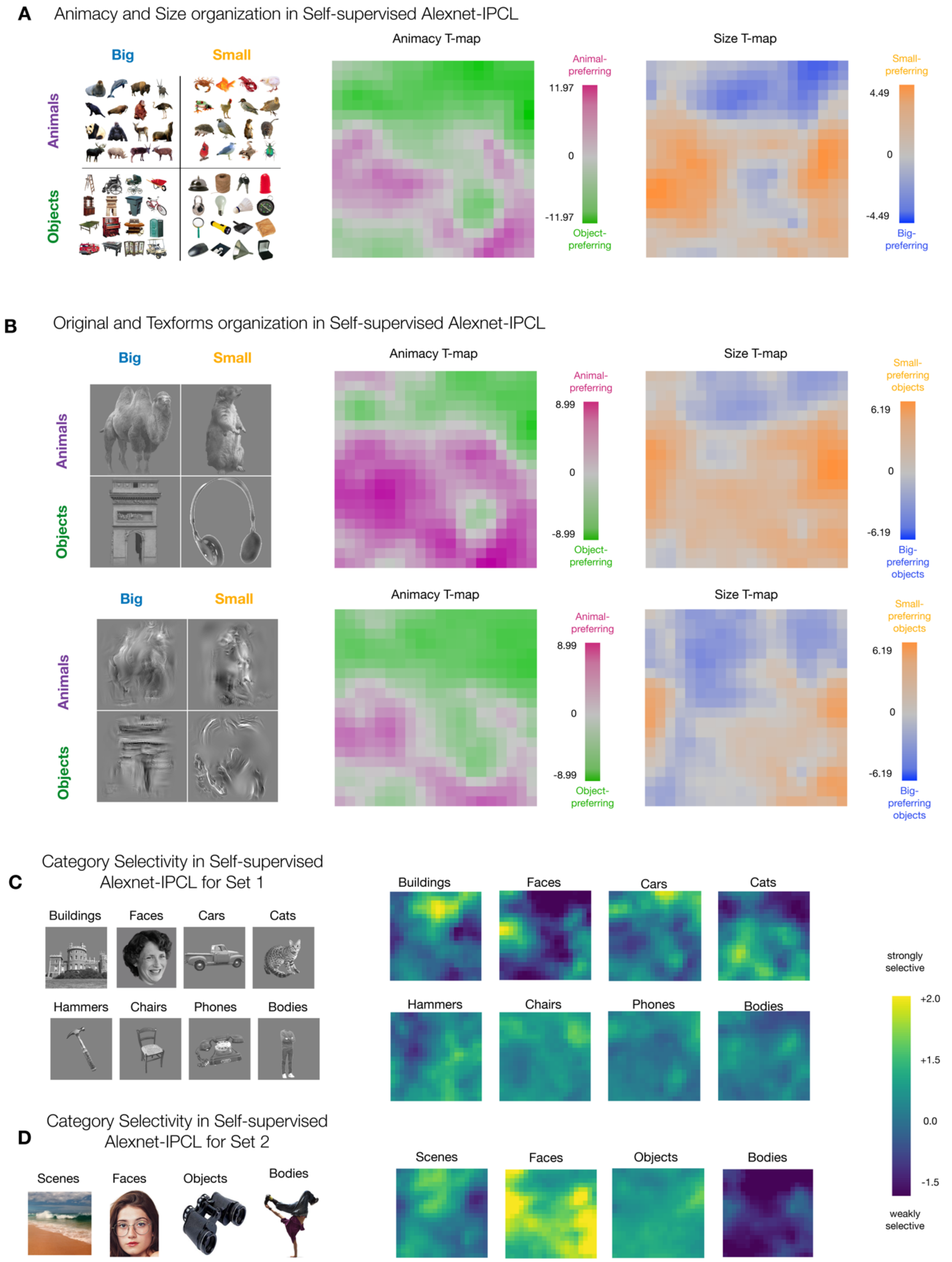

Fig. S13. Topographic motifs in a self-supervised DNN (IPCL).
(A) Animacy and size distinctions on the SOM for images containing animals and objects of different sizes (left). Each unit on the SOM is colored by its response preference (t-statistic) to either animal

or object images (center) and to images of either big or small entities (right). (B) The same three subplots, here shown for grayscaled original and texform images. (C, D) Example images from each category in Set 1 and 2 and the corresponding category-selective maps computed using the d-prime metric.

Supplementary Analysis – Pixel Space Representation

In Supplementary set of analyses, we examined whether the distinctions for animate vs inanimate and big vs small objects emerge in a pixel space representation. That is, without the deep-neural network untangling that has been learned in a pre-trained Alexnet, do we already see these distinctions in pixel space, and to what degree?

To test this, we fit an SOM to the pixel space of the validation images of the ImageNet database, yielding an input matrix of 50,000 images x 150,528 dimensions (reflecting the RGB encoding of each image in 224 pixels * 224 pixels * 3 channels). The pixel-SOM shape was automatically set to 14 x 28 units. This is the largest map size that is <= 400 units, that preserves the ratio of the first two eigenvalues of the sample of 400 images). Next, we probed the pixel-SOM with the same animal and object images (Konkle & Caramazza, 2013 *(9)*). The results are shown in **Supplementary Figure 4**.

We find that in the preference maps for the pixel-SOM, most units had a stronger activation on average to animal images (**Supplementary Figure 4A**). When we quantify the separability between animals and object images with an independent 2-sample t-statistic, however, we find only weak separability between these two classes of images, as evident in the plotted t-maps (e.g., the average absolute value of the t-statistics across the map is $|t|=1.29$, the max is $t=2.38$). For comparison, when we compute the t-maps over the relu7 space, we see clearer separability between animal vs object image responses in each unit (average $|t|=6.54$, max $|t|=16.12$; (**Supplementary Figure 4B**). Thus, the animal and object images are dramatically more entangled in the pixel space than in the trained relu-7 space.

Next, considering the big vs small distinction, we find that in the pixel-SOM, most units have a stronger activation on average to small objects (**Supplementary Figure 4C**). However, as before, there is only weak separability between big vs small entities (average $|t| = 1.98$, max $|t|=2.37$). In contrast, the relu7 space t-maps show clear separability between animal and object images (average $|t|=3.25$, max $|t|=11.97$; (**Supplementary Figure 4D**). Thus, images of big and small entities are also dramatically more entangled in the pixel space than in the trained relu-7 space.

In doing these analyses, we noticed the localizer images were dramatically out-of-distribution compared to the ImageNet validation images in pixel space (e.g. the localizer images are on a white background, affecting many of the pixel dimensions). So, in a exploratory analysis, we additionally trained a pixel-SOM directly on the localizer image set (240 images x 150,528 dimensions, yielding a 20x20 localizer-pixel-SOM). And, we probed it with the same images. We again find the animal preferences and small preferences were found across the entire map, and again that t-maps showed minimal separability between these classes (animacy: average $|t|=2.19$, max $|t| = 2.91$, size: average $|t| = 2.94$, max $|t| = 3.22$). In contrast, an SOM trained only on these 240 images as they are embedded in the relu7 space (240 x 4096) showed clear animacy and object size organizations (animacy: average $|t|=7.59$, max $|t|$ - 17.2; size: average $|t| = 3.64$, max $|t| = 8.04$). Thus, even in the context of just the localizer images, the animacy distinction is untangled in the pixel space of this image set, which has become untangled by the relu7 stage of the deep neural network.

A further question we wondered was whether is it always the case that animals and small object images a stronger preference in pixel space, or are these preferences a property of this particular set of 240 color images? We next probed the ImageNet-Pixel-SOM with the gray-scaled versions of

these same images, as well as the texforms (stimulus set from Long et al. *(11)*). Here, units showed both animate and inanimate preferences, and big and small preferences, on average, though again with all t<s very low. Thus, this additional exploratory analysis implies that it is not a general case that animals and small objects are more extreme in pixel space.

Taken together, these analyses demonstrate that the distinctions between animals and objects and big and small entities are very entangled in pixel space. And, the reformatting of image information through hierarchical stages of a deep neural network is critical to see these distinctions emerge.
